# Structural Optimization and Fatigue Performance Evaluation of Balloon-Expandable Aortic Valve Stents

**DOI:** 10.3390/ma19194083

**Published:** 2026-09-24

**Authors:** Tao Cai, Chaoqun Zhao, Gang Fang

**Affiliations:** Department of Mechanical Engineering, Tsinghua University, Beijing 100084, China

**Keywords:** aortic valve stent, structural design optimization, in vitro bench test, finite element simulation

## Abstract

Transcatheter aortic valve implantation (TAVI) is a critical therapy for aortic stenosis, but most existing valve stents are designed for Western patients, whose aortic root anatomy differs from Chinese patients characterized by smaller annulus diameters, heavier calcification, and a higher prevalence of bicuspid valves. To address this mismatch, we designed a balloon-expandable aortic valve stent tailored for Chinese patients and systematically evaluated its performance. Four structural schemes were considered; a diamond–hexagonal composite mesh was selected based on radial strength, expansion uniformity, anchoring stability, sealing, and coronary access. Material properties of MP35N were obtained via tensile tests, and finite element models simulated crimping, recoil, and expansion. Among three end-corner geometries, the U-shaped design showed superior mechanical behavior with lower stress/strain concentrations. For stents crimped to 6.5 mm, maximum equivalent stress ranged from 1030 to 1076 MPa and plastic strain from 0.221 to 0.259; upon expansion to 20–29 mm, stress reached 1417–1493 MPa and strain 0.447–0.544, with strain concentrated at the corner ends. Simulated recoil matched experimental results within 7% relative error. Accelerated fatigue testing (4 × 10^8^ cycles) of the bare stents revealed no fractures, displacements, or surface damage. These findings demonstrate that the optimized bare stent structure exhibits good mechanical feasibility, durability, and improved anatomical compatibility for Chinese TAVI patients, providing a promising device solution for domestic clinical application.

## 1. Introduction

In the context of global population aging, the incidence of cardiovascular disease continues to increase, and aortic stenosis has become one of the major diseases that seriously threaten the health of the elderly [1]. Aortic stenosis can cause increased left ventricular outflow tract resistance, leading to heart failure, syncope, and other clinical symptoms, seriously affecting the quality of life and life expectancy of patients [2]. Epidemiological studies have shown that the incidence of aortic stenosis in people over 65 years old is about 2% to 7%, and it shows a significant upward trend with age [3]. At present, the main treatments include drug therapy, surgical aortic valve replacement (SAVR), and transcatheter aortic valve implantation (TAVI) [4]. Among them, TAVI does not require traditional thoracotomy and can be transported to the aortic valve area through vascular access to complete the release [5]. It has the characteristics of less trauma and faster postoperative recovery and has become an important method for interventional treatment of aortic stenosis [6].

The transcatheter aortic valve system is mainly composed of a valve and a delivery system. The valve mainly includes a stent, a biological valve leaflet, and a sealed skirt. Each component performs different functions, as shown in Figure 1.

Its structural performance directly affects crimping transmission, release expansion, apposition stability, and long-term durability. The artificial valve leaflets are periodically opened and closed under the action of transvalvular pressure gradient to realize one-way blood flow. The kinematics of artificial valve leaflets directly affects the hemodynamic performance, such as transvalvular pressure gradient, effective orifice area, and regurgitation. The sealing skirt is mainly used to improve the bonding state between the stent and the original annulus and reduce the risk of perivalvular leakage. The delivery system is responsible for the compression loading, catheter delivery, positioning, and release functions of the artificial valve. For balloon-expandable prosthetic valves, the metal stent needs to undergo compression, delivery, balloon expansion, unloading recoil, and long-term pulsating cyclic load after implantation in turn during the implantation process. The unloading recoil and long-term pulsating cyclic load after implantation are accompanied by obvious geometric nonlinearity, local plastic deformation, and stress concentration. Therefore, the mesh configuration, strut size, and joint geometry of the stent not only determine its expansion and recoil characteristics, but also directly affect the structural integrity and long-term fatigue reliability. Based on this, this study mainly investigates the structural design and mechanical properties of a balloon-expandable artificial aortic valve metal stent, and the hemodynamic performance of the valve leaflet is not the focus of this study.

In addition, there are some differences in aortic valve anatomy and lesion characteristics between Chinese and Western patients [7]. The overall size of the aortic annulus in Chinese patients is relatively small, with an average diameter of about 21.3–23.1 mm [8], and about 60% of patients have an annulus diameter of less than 23 mm [9]. The proportion of bicuspid aortic valve (BAV) is relatively high [10], and it is accompanied by more complex leaflet calcification characteristics [11]. The differences in annulus size, valve morphology, and calcification distribution will change the local constraints in the stent release process [12], and further affect the radial strength, expansion morphology [13], anchoring stability, and perivalvular sealing effect of the stent [14]. Therefore, it may be difficult to fully meet the structural adaptation needs of Chinese patients with different annulus sizes and complex lesions [15] by directly using the stent size and mesh configuration based on the anatomical characteristics of other populations.

Based on the above issues, this study focuses on the design, manufacture, and verification of a balloon-expandable artificial aortic valve stent structure. Firstly, different mesh configurations of the valve stent were proposed according to the anatomical size of the aortic valve and clinical functional requirements of Chinese patients, and the stent scheme was screened by multi-index evaluation [16]. Subsequently, a finite element model was established for the corner structure of the stent and different specifications of the stent, and the stress, plastic strain, and geometric deformation characteristics during the process of crimping, recoil after crimping, expansion, and recoil after expansion were analyzed. On this basis, the stents of the MP35N alloy were fabricated from the tubes by a precise laser cutting process. The surface quality of the stents was improved by sandblasting to remove burrs, electrochemical polishing, and passivation, and the influence of cutting defects and surface micro-cracks on the structural performance was reduced. Finally, the finite element simulations were verified by the deformation tests of the stents. Accelerated fatigue tests of the stents showed that the fabricated stents were undamaged and qualified. The purpose of this study is to clarify the relationship between stent geometry and local mechanical response, and to provide a basis for the structural design and serial specification development of a balloon-expandable artificial aortic valve stent.

## 2. Stent Structure Design

### 2.1. Structural Scheme

Based on the characteristics of small annulus size, high proportion of bicuspid aortic valve and severe calcification of valve leaflets in Chinese patients with aortic stenosis, this study takes suitability for small annulus, radial support ability, crimp-and-delivery performance, expansion uniformity, anchoring stability, sealing and apposition performance, and coronary access as the main objectives of stent structure design. Among them, the average diameter of the aortic annulus in Chinese patients is about 21.3–23.1 mm, and about 60% of the patients’ aortic annulus diameters are less than 23 mm; the proportion of bicuspid aortic valves is about 40–50%, and severe calcification is mostly concentrated at the leaflet level.

Based on these anatomical features, we proposed four preliminary stent design schemes, as shown in Figure 2. In the first scheme, a large hexagonal mesh with equal pitch is exhibited, and the radial strength, coronary perfusion and PCI operation space are considered through a larger cell opening. In the second scheme, a diamond-shaped cross mesh is employed together with continuous oblique support rods to enhance the circumferential constraint and radial load-bearing capacity, with the primary aim of improving the stent’s anchorage stability. In the third scheme, features uniformly distributed small hexagonal meshes and improves the expansion uniformity and wall-apposition performance of the stent by increasing the number of cells. In the fourth scheme, a diamond–hexagonal composite mesh with gradual partition variation is employed. Dense small-sized cells were arranged near the annular end to enhance the radial strength, anchoring performance and sealing ability. A large-opening structure was used near the aortic end to reserve space for coronary perfusion and re-intervention. In the intermediate region, gradual mesh variation enabled a smooth transition of load and deformation.

The balloon-expandable aortic valve stent needs to meet the requirements of crimping transmission, radial strength, expansion forming, anchoring and adherence, coronary perfusion and long-term fatigue at the same time. There is a certain restriction relationship between various properties [17]. Therefore, the stent structure cannot simply improve a certain performance. The design goal is to achieve multi-objective coordination through a reasonable combination of mesh size, mesh shape, strut size and regional distribution.

The cell size of the stent is a critical geometric parameter that influences the mechanical properties of the structure. As the mesh size increases, the number of struts and connecting nodes per cell circumferential length decreases, thereby enhancing the overall flexibility of the stent. This facilitates crimping and folding, reduces metal coverage, and provides larger open spaces for leaflet expansion, coronary perfusion, and subsequent catheter passage. However, excessively large meshes reduce the effective contact area between the stent and the annulus, leading to decreased local radial strength and impaired wall-apposition continuity. On the contrary, smaller and denser meshes increase the circumferential load paths and contact area, which benefits the radial strength, anchoring and sealing capacity of the inflow area. Nevertheless, this also elevates structural constraints and metal coverage during the crimping process. Therefore, a single-size mesh finds it difficult to simultaneously balance deliverability and support stability.

The shape of the cell also influences the deformation mechanism of the stent. The diamond-shaped cells primarily rely on angular changes to achieve radial compression and expansion, providing a clear geometric deformation path that makes them suitable for transition regions requiring large radial deformation. However, radial expansion is accompanied by significant axial dimensional changes, necessitating careful attention to stent foreshortening and localized deformation concentration at the connected points. The hexagonal cell has good circumferential continuity, which can form multiple interconnected bearing paths, which helps maintain post-expansion roundness and the stability of radial strength. Nevertheless, excessively dense meshes increase structural stiffness, which is detrimental to achieving a low profile. Therefore, the diamond-shaped and hexagonal structures are not simply superior or inferior to each other; rather, they place different functional emphases on compliant deformation, radial strength, structural openness and expansion stability.

In addition to the cell size and shape, the strut width, wall thickness and connection fillet are also key parameters that determine the performance of the stent. Increasing the width or wall thickness of the strut can improve the local bearing capacity and radial strength, but it will increase the crossing profile. Although reducing the strut size benefits conveying and compliant deformation, it reduces the radial strength and fatigue safety margin. It is easy to produce obvious geometric mutation and stress concentration when the fillet at the joint is too small. Therefore, a continuous arc transition is needed to make the bending deformation generated in the process of crimping and expansion transfer gradually over a large range.

Therefore, a partitioned diamond–hexagonal composite mesh structure was determined for the valve stent. This design no longer assumes uniform geometric characteristics across the entire support but instead applies differentiated configurations according to the functional requirements of different axial regions. The inflow area features relatively small and densely arranged meshes to improve contact continuity and radial restraint between the stent and the annulus, thereby meeting the requirements for anchoring and sealing. In the middle section, transition cells that combine diamond and hexagon geometries are introduced, enabling the stent to coordinate radial and axial deformation through angular changes in the cells during the crimping and expansion, while reducing abrupt stiffness transitions between regions. In the outflow region, larger open cells are used to reduce metal coverage and reserve space for valve leaflet motion, coronary blood flow, and subsequent catheter and guide-wire manipulation. Smooth connections between meshes of different sizes allow the stent to transition gradually from a high support zone to a high-openness zone.

According to the principle of structural mechanics, corner locations typically experience severe stress concentration. Accordingly, three end-corner configurations for the stent were investigated: U-shaped, Y-shaped, and C-shaped, as shown in Figure 3. In the U-shaped design, a large-radius arc to distribute bending deformation over a longer path is used, minimizing stress concentration and maximizing flexibility. The Y-shaped design forms a rigid converging node with bifurcated struts, enhancing radial strength and anchoring stability at the cost of higher local stress. The C-shaped design adopts an intermediate curvature to balance compliance and stiffness.

### 2.2. Stent Specifications

For the fourth scheme obtained by screening, in order to cover the implantation needs of patients with different aortic annulus sizes, this study was based on the above-mentioned aortic annulus size distribution of Chinese patients and combined with the serial design principle of balloon-expandable valve stents; thus, stent nominal diameters were stratified into grades. Considering that the aortic annulus of Chinese patients is generally small, and there are some differences in the size of the annulus between different patients, it is gradually extended to the larger annulus range based on small-size adaptation. Finally, four specifications of CDT07-20, CDT07-23, CDT07-26 and CDT07-29 were set up, and the nominal outer diameters of the corresponding stents were 20, 23, 26 and 29 mm, respectively. The adjacent specifications exhibit a size gradient of 3 mm, which can not only form a continuous size coverage but also avoid the complexity of the product series caused by excessive specification division, considering the patient’s anatomical adaptability and engineering serialization design requirements. The four specifications exhibit the same diamond–hexagonal composite mesh configuration and node form, and the geometric size is adjusted only according to the target diameter, to further compare the mechanical response of different stent specifications under crimping, expansion, recoil and fatigue load. The overall model of the stent and the symmetric model for finite element analysis are shown in Figure 4. The stent model exhibits a diamond–hexagonal composite mesh structure, with a wall thickness of 0.45 mm and a strut width of 0.35 mm. The CDT07-20 specification (outer diameter 20 mm) was selected as a typical model.

## 3. Finite Element Analysis

### 3.1. Material Properties

This study simulated the complete deformation process of the valve stent from the initial state through external compression, recoil after crimping, expansion and recoil after expansion. Since the stiffness of the crimping tool and the expansion tool is much larger than that of the valve stent, and the research focus is on the elastic–plastic deformation of the stent under contact load, the crimping tool and the expansion tool are defined as rigid bodies in the finite element model, regardless of the elastic deformation of the tools. The reference points are established on the crimping tool and the expansion tool, respectively, and the tool surface is rigidly coupled with the corresponding reference point. The radial displacement is applied to the reference point to control the crimping and expansion process, to avoid the influence of tool deformation on the calculation results of the stent. All finite element simulations were performed using Abaqus/Standard 2014 (version 6.14). The calculations were conducted on a computer equipped with a 32-core Intel Xeon Platinum 8375C processor (Intel Corporation, Santa Clara, CA, USA) and 128 GB of RAM.

In the finite element analysis, the material parameters and loading path of the stent need to be determined by combining the material test [18] with the simulation study of the existing valve stent. Considering the characteristics of aortic valve disease and the requirements of mechanical properties, cobalt–nickel–chromium alloy (MP35N) was selected as the stent material [19]. MP35N is a high-strength multi-phase cobalt-based superalloy with high tensile strength, good ductility, toughness, and corrosion resistance, which is suitable for cardiovascular interventional instruments [20]. In order to obtain the basic mechanical properties of the stent material MP35N and provide reliable material parameters for the finite element analysis of the stent, the tensile deformation and failure characteristics were studied by uniaxial tensile testing at room temperature. The uniaxial tensile test of the material was carried out three times, and the repeatability was very good. Figure 5 shows the true stress–strain curve of MP35N after averaging treatment. According to the results of the uniaxial tensile test, the elastic modulus of MP35N is 2.70 × 10^5^ MPa (270 GPa), and Poisson’s ratio is 0.30. The yield stress is 399.3 MPa. The true stress–plastic strain data after yielding are obtained by using the stress–strain curve of Figure 5.

### 3.2. Meshing

To ensure computational accuracy, the C3D8R element in Abaqus was adopted for the finite element modeling of the stent [21]. C3D8R is an eight-node linear hexahedral solid element with reduced integration. The reduced-integration formulation effectively avoids shear locking that would otherwise occur in fully integrated linear hexahedral elements under bending or large-deformation conditions, while also improving computational efficiency by employing a single integration point. These features make C3D8R particularly suitable for large-deformation analyses in which bending and distortion are significant. In addition, appropriate hourglass control is applied to suppress non-physical zero-energy deformation modes. Four element layers were defined across the stent thickness. Taking the CDT07-20 stent as an example, the total number of finite elements is 19,064. The crimping tool and the expansion tool were modeled using the S4R mesh element in Abaqus, which is a general-purpose shell element suitable for both thin and thick shells. The mesh after assembly is shown in Figure 6.

### 3.3. Boundary Conditions

To simulate the complete process of valve stent crimping, the outer surface of the stent was placed in contact with the inner surface of the crimping tool, while the inner surface of the stent was placed in contact with the outer surface of the expansion tool. Normal contact behavior was defined as ‘hard contact’, which allows the contacting surfaces to separate after unloading. Tangential contact was modeled using a penalty-based friction model with a friction coefficient of 0.15. In addition, self-contact was defined for the potential contact regions of the stent itself to prevent mutual penetration of the struts during large deformation.

Considering the periodic repetition characteristics of the stent along the circumferential direction, a one-third circumferential model was used for computation. A periodic symmetrical constraint is applied to the 120° circumferential cutting boundary on both sides of the model, so that the radial displacement, axial displacement and rotation of the corresponding nodes on both sides meet the periodic consistency relationship, to ensure that the 1/3 model can represent the circumferential continuous deformation of the complete support during the crimping and expansion process. At the same time, the characteristic nodes in the middle of the stent are selected to limit the axial rigid-body translation, and minimum essential constraints were applied to suppress global rigid-body rotation, to avoid the influence of additional constraints on the normal radial deformation of the stent.

The crimping process exhibits the loading method of active movement of the crimping tool and passive deformation of the stent. The crimping tool is initially located on the outside of the stent, and the radial inward displacement is applied through its reference point, so that the crimping tool shrinks uniformly along the circumferential direction, and the outer diameter of the stent is gradually compressed to 6.50 mm. During the crimping process, the radial displacement of the stent itself is not directly applied, and its deformation is completely caused by the contact between the crimping tool and the stent. After the target crimping diameter is reached, the loading state is maintained, and then the radial constraint of the crimping tool is removed, so that the stent can recoil freely under the action of material elastic recovery, and the residual stress, plastic strain and recoil outer diameter after crimping are recorded.

In the expansion stage, the expansion tool undergoes active radial movement while the stent expands passively. The expansion tool moves radially outward from inside the stent and applies a uniform expansion effect to the stent’s inner surface, thereby expanding stents of different specifications to the target diameters of 20, 23, 26, and 29 mm, respectively. Once the target expansion size is reached, loading is stopped, and the radial displacement constraint of the expansion tool is removed, allowing the stent to expand freely and undergo recoil. Geometric nonlinearity is activated throughout the entire analysis to account for large deformation, changes in contact state, and the elastic–plastic material response of the stent during both the crimping and expansion processes [22].

## 4. Results of Finite Element Simulations 

### 4.1. Mechanical Response of End-Corner Structures During Crimping

Figure 7, Figure 8 and Figure 9 show the stress and strain distributions of the three end-corner configurations (‘U’ shape, ‘Y’ shape, ‘C’ shape) of the stent under compression, respectively. The maximum von Mises equivalent stresses of the ‘U’-shaped, ‘Y’-shaped, and ‘C’-shaped stents are 1001 MPa, 1029.31 MPa, and 1037.89 MPa, respectively, and the corresponding maximum equivalent plastic strains are 0.205, 0.237, and 0.267, respectively. Through the comparative analysis of the maximum stress and strain, the ‘U’-shaped structure prossesses the best performance among the three types of stent end-corner structures. Compared with the C-shaped configuration, the U-shaped configuration reduces peak stress by 3.55% and peak plastic strain by 23%, indicating that the ‘U’-shaped structure obtains better performance than other configurations.

### 4.2. Full-Process Deformation Analysis of Stents with U-Shaped Corners

Taking the stent CDT07-20 as an example, the deformation process is mainly divided into four stages. The specific operation is as follows:

(1) Crimping process operation: During crimping, stepwise loading was applied to impose uniform radial displacement on the stent via the crimper, so that the outer diameter of the stent was reduced from the initial 20 mm to 19.36 mm, 15.82 mm, 11.25 mm, and finally reached 6.50 mm. During each step of the crimping process, the stress state and deformation of the stent were continuously monitored, and the stress and strain distribution data under different outer diameters were recorded.

Figure 10 and Figure 11 show the von Mises equivalent stress distributions and equivalent plastic strain distributions of the valve stents during crimping. Plastic deformation first initiates at the curved corner joints of the stent. As crimping proceeds, regions of plastic deformation progressively expand. From the perspective of the final stage of compression, the plastic deformation area is mainly located at the arc connection of the stent corner, and the deformation at the linear connection is relatively small. The maximum equivalent stress of the stent is 1030.5 MPa, and the maximum equivalent plastic strain is 0.221.

(2) Recoil process after crimping: After the stent was crimped down to an outer diameter of 6.50 mm, this crimped configuration was held for a dwell period, and then the pressure of the crimping device was gradually released. During the pressure release process, morphological evolution was continuously monitored, and the final outer diameter after crimp recoil was recorded, and the release of internal residual stress during the recoil process was evaluated.

After the compression, the crimping device was removed, and the stent recoiled. With the disappearance of elastic deformation in the recoil process, the stress applied to the stent decreases continuously. The residual stress distribution before and after compression and recoil is shown in Figure 12. The maximum equivalent stress of the stent after recoil is 479.5 MPa. At the same time, the outer diameter of the stent after compression and recoil is 7.30 mm (Figure 13).

**Figure 12:**
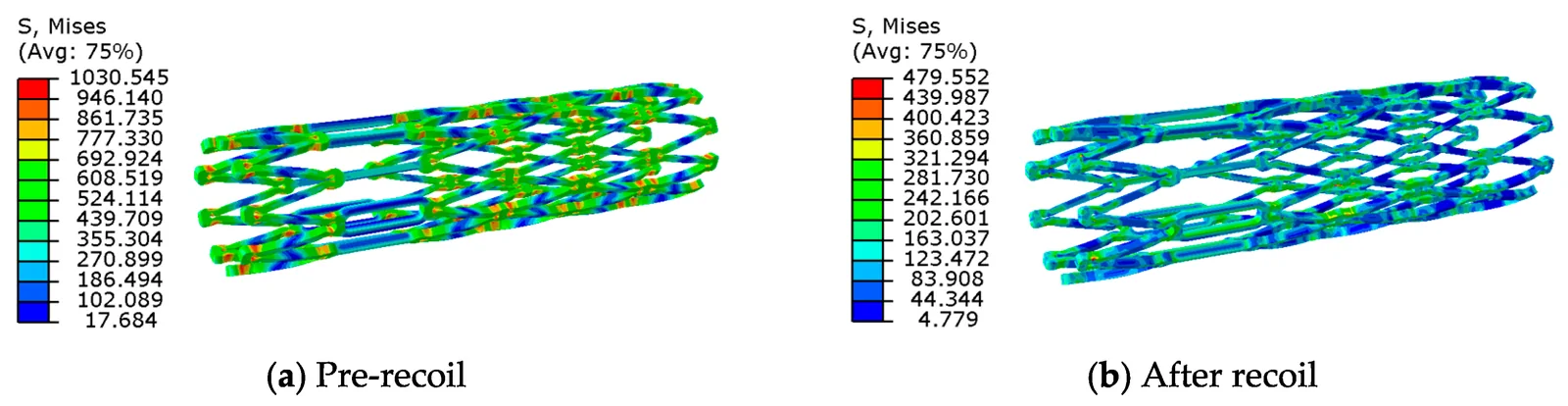
The residual stress distribution (MPa) before and after crimping recoil of the valve stent.

**Figure 13:**
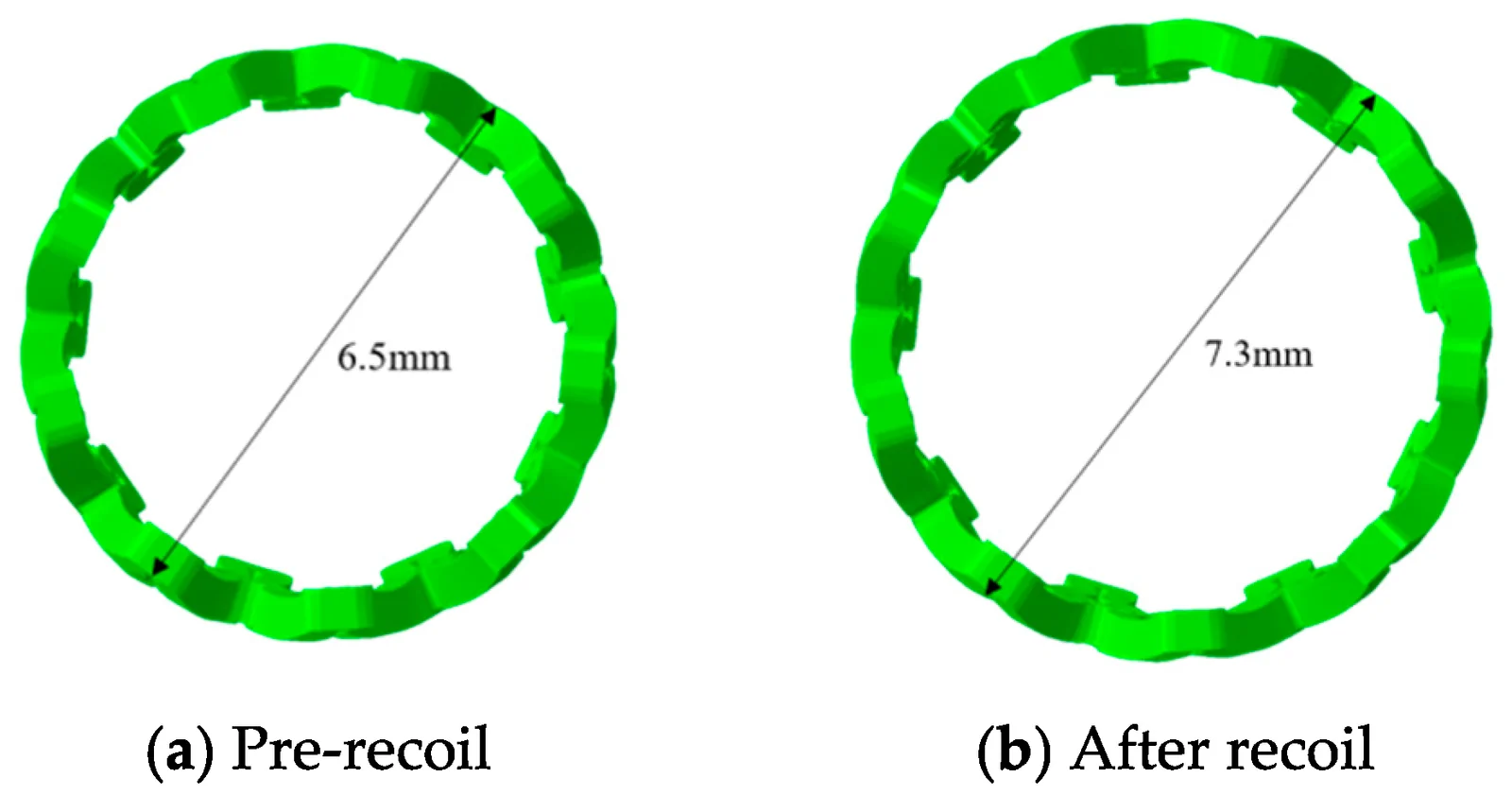
The outer diameter of the valve stent before and after crimping recoil.

(3) Expansion process: After crimping and recoiling, the expander tool was placed inside the stent, and the stent was gradually expanded by adjusting the expander tool. The outer diameter of the stent increased from 7.80 mm, 11.96 mm, and 15.67 mm to 20.00 mm. During the expansion process, the expander tool was kept uniform to avoid excessive local stress, and the deformation characteristics of the stent in each expansion stage were recorded.

After crimping and recoiling, the stent expands under the expander tool. Figure 14 and Figure 15 are the von Mises equivalent stress distribution and equivalent plastic strain distribution of the valve stent during expansion. During the expansion of the stent, the radial displacement increases; the stent exhibits noticeable axial foreshortening, and the position with larger equivalent stress is still at the arc connection of the stent corner. At the end of expansion, the maximum equivalent stress of the stent is 1417.3 MPa, and the maximum equivalent plastic strain is 0.447.

(4) Recoil process after expansion: Once expanded to the target outer diameter of 20.00 mm, the expanded state was held for a dwell time, followed by retraction of the expander tool. In this process, the recoil behavior and residual stress changes of the stent after losing the support were monitored, and the final outer diameter after recoil was recorded.

After the end of the expansion, the expander tool was removed, the elastic deformation of the stent was restored, and the recoil phenomenon occurred again. Figure 16 shows the residual stress distribution before and after the expansion of the valve stent. A large amount of stress is released during the recoil process, and the maximum equivalent stress after recoil is 601.3 MPa. At the same time, the outer diameter of the stent after expansion and recoil is 19.50 mm (Figure 17).

### 4.3. Mechanical Response of Multi-Specification Stents

For the other three specifications CDT07-23, CDT07-26, and CDT07-29, the crimping, crimping recoil, expansion, and expansion recoil tests were carried out according to the CDT07-20 operation process. Among them, the target crimping outer diameter of CDT07-23 is 6.50 mm, and the target expansion outer diameter is 23.00 mm. The target crimping outer diameter of CDT07-26 is 6.50 mm, and the target expansion outer diameter is 26.00 mm. The target crimping outer diameter of CDT07-29 is 6.50 mm, and the target expansion outer diameter is 29.00 mm. In each stage of operation, a step-by-step loading/unloading method is exhibited to ensure the accuracy and stability of data acquisition.

Table 1 lists the maximum equivalent stress, the maximum equivalent plastic strain, and the outer diameter after recoil of the four kinds of stents in the crimping, crimping recoil, expansion, and exnsion recoil stages.

## 5. Results of Experiments

### 5.1. Comparison of Tested and Simulated Stent Deformations

To obtain a solid stent that is consistent with the geometric parameters of the finite element model, an MP35N alloy pipe is selected as the raw material and manufactured in accordance with design drawings for the CDT07-20 and CDT07-26 stents. Firstly, the precision laser cutting process is used to form a predetermined diamond–hexagonal composite mesh structure on the surface of the pipe, and the width of the supporting rod, the connection node, and the end-corner contour after cutting are checked. Since there may be a small amount of slag, burrs, and heat-affected zones (HAZs) on the surface of the stent after laser cutting, further cleaning, deburring, and electrochemical polishing are carried out to reduce the surface roughness of the support rod and improve the surface quality of the joint area. Subsequently, passivation treatment was carried out to improve the corrosion resistance of the stent surface. After the processing is completed, the overall outer diameter, strut integrity, mesh shape, and connection area of the stent are checked, and the samples with obvious cutting defects or geometric deformation are eliminated to ensure that the solid stent used for crimping and expansion tests has good consistency with the design model.

On this basis, the solid stent was successively crimped and balloon-expanded, and the structural states of the initial forming, crimping, and expansion were recorded respectively for comparison with the corresponding stages of the finite element model of the same specification. Figure 18 and Figure 19 give the solid and simulation deformation results of the CDT07-20 and CDT07-26 stents, respectively. In general, the overall contour, mesh deformation direction, and radial size variation of the two types of stents in each stage are in good agreement, indicating that the established finite element model can accurately reflect the macroscopic deformation characteristics of the stent in the process of pressing and expansion. After the initial forming, slight warping occurs at both ends of the solid stent, which is mainly related to the release of residual stress after laser cutting and the elastic recovery of the stent itself. After crimping, the tested stent and the simulated stent form a relatively uniform and compact shrinkage configuration, and the mesh folding position is basically consistent with the overall shape. After expansion, the main area of the stent can be fully expanded, the mesh structure is evenly distributed, and the overall shape is consistent with the simulation results. The end of the tested stent showed a certain ‘dog bone’ effect, indicating that the end of the balloon was expanded before the middle during the balloon expansion. The above results verify the ability of the finite element model to predict the overall crimping deformation process and expansion morphology of the stent.

### 5.2. Stent Expansion/Recoil Verification

To verify the ability of the finite element model to predict the recoil behavior of the valve stent after crimping and balloon expansion, four types of stents, CDT07-20, CDT07-23, CDT07-26, and CDT07-29, were selected to carry out crimping recoil and expansion recoil tests. The initial outer diameter of the stent was measured before the test, and then the stent was crimped to the specified loading diameter by the crimper. The crimper constraint was removed, and the outer diameter was measured after the stent shape was stable, and the crimping recoil was calculated. After that, the stent was expanded to the target diameter with a matching balloon. After the balloon was depressurized and removed, the stable outer diameter of the stent was measured again to obtain the expansion recoil. The test conditions, measurement positions, and stabilization time of each specification are consistent, and the test results are compared with the finite element results. To reduce the accidental error caused by a single experiment, the average value was taken after 10 repeated tests of each specification. The results are shown in Table 2.

From the results of Table 2, as the stent specification increases from 20 mm to 29 mm, the amount of crimping recoil and expansion recoil generally rises. In terms of crimping recoil, the simulated value of simulation increased from 0.80 mm to 1.30 mm, the tested value increased from 0.78 mm to 1.23 mm, and the relative error was 2.56~5.88%. In terms of expansion recoil, the simulated value increased from 0.50 mm to 0.80 mm, the value of tested increased from 0.47 mm to 0.86 mm, and the relative error was 5.26~6.98%. Among them, the recoil prediction accuracy of the 20 mm specification is the highest, and the error is only 2.56%. The expansion recoil error of the 23 mm specification is the smallest, at 5.26%. The absolute error under all working conditions is not more than 0.06 mm, and the relative error is less than 7%, which is basically consistent with the change rule. It shows that the established finite element model can accurately predict the radial recoil behavior of different specifications of stents after crimping and expansion unloading. The deviation of expansion recoil of the 29 mm specification is relatively large, which may be related to the dispersion of large-size stent materials, the uniformity of balloon expansion, and the actual assembly error, but it is still in the acceptable range.

### 5.3. Fatigue Performance Evaluation of Valve Stent

According to the method specified in YY/T 0808-2010 [23], the fatigue test was performed in phosphate-buffered saline (PBS) at (37 ± 2) °C [24] as shown in Figure 20. The frequency of the fatigue tester was 20 Hz with a pressure range of 80–160 mmHg [25]. The following picture shows the in vitro fatigue test system of the valve stent. The main body consists of two BOSE-brand moving-magnet linear motors, symmetrically arranged on both sides of the electroforce vascular stent fatigue testing system (Bose Corporation, Eden Prairie, MN, USA), providing pulse pressure loading for the test circuit through reciprocating motion. The central area of the device is the test core: the simulated vessel is horizontally fixed by the simulated vessel mounting clamp, and the valve stent to be tested is implanted in the lumen; the upper left enlarged view clearly shows the valve stent in the loading zone of the valve stent. The stent is subjected to periodic pulse pressure in simulated blood vessels to accelerate the assessment of its long-term fatigue durability. The laser diameter-measuring module is equipped around the simulated vascular loading valve stent to monitor the diameter change of the simulated blood vessel in the pressure cycle in real time.

The fatigue durability test waveform is shown in Figure 21. The typical cycle waveforms intercepted in the stable operation stage of the fatigue test are given in the figure. Overall, each signal shows relatively stable cycle repetition characteristics in multiple consecutive cycles, and no obvious baseline drift or amplitude mutation occurs, indicating that the loading process in the test stage has good repeatability. Disp. (dark blue curve) is the volume displacement, which represents the volume output signal of the reciprocating motion of the servo piston of the fatigue testing machine, and is the driving source of the whole hydraulic pulsation system. The curve shows a regular periodic change, indicating that the test device can continue to complete the reciprocating motion during the loading process. Ext Press (green curve) is the measured pressure in the cavity, which represents the real-time hydraulic pressure collected by the first pressure sensor in the cavity to simulate the alternating systolic/diastolic blood pressure of the human body. The curve mainly fluctuates in the range of about 116.9–122.1 mmHg. Although there are some local high-frequency fluctuations, the overall variation range of each cycle is similar, and there is no continuous increase or decrease trend. Press (red curve) is the system control pressure, which represents the feedback pressure of the closed-loop control of the testing machine. It is used to automatically adjust the piston stroke of the system to ensure that the overall pressure is stably maintained in the set range of 80~160 mmHg. The pressure and displacement curves maintained stable phase correspondence, and the peak and valley values are basically the same in each cycle, indicating that the cyclic loading process is continuous and stable. The diameter (purple curve) simulates the outer diameter of the blood vessel, which represents the optical sensor to monitor changes in the outer diameter of the simulated blood vessel in real time, and directly reflects the expansion and contraction deformation state of the stent under alternating pressure. It is the core parameter to judge the fatigue failure, plastic relaxation, and fracture of the stent. The curve changes periodically in the range of 37.21–39.75 mm, and maintains good synchronization with the loading process, indicating that the stent can produce repeated radial compression and recoil under cyclic loading. It can be seen from the curves that during the test period, each monitoring signal maintains a stable periodic response, and no obvious abnormal jump or cumulative offset is found, which provides basic data support for the subsequent long-term cycle durability evaluation.

After 4 × 10^8^ cycles, no obvious displacement was observed in all valve stents in the simulated blood vessels (Figure 22), and the stent structure remained intact without evidence of fracture.

## 6. Discussions

The core of this study is to establish a balance between radial strength, crimping and delivery, expansion uniformity, anchorage adherence, coronary artery channel, and long-term fatigue reliability. Based on the comprehensive evaluation of clinical needs, a diamond–hexagonal composite mesh scheme was selected. Its merit lies in presenting a partitioned-function structure to spatially distribute different mechanical requirements: the dense mesh near the annulus end bears the main radial strength and anchoring effect, the larger opening cell at the outflow area is conducive to retaining the coronary artery channel, and the intermediate gradient area bears the stiffness transition function. Compared with the single repetitive mesh, the advantage of the composite configuration should be understood as the adjustment of the load-bearing and deformation modes of different regions through geometric partitions, rather than the pursuit of uniform maximum stiffness of the entire stent.

The corner-end is the main deformation cell in the process of crimping and expansion of the stent, and its geometric shape directly affects the load transfer mode. The finite element results show that the maximum equivalent stresses of U-shaped, Y-shaped, and C-shaped structures are 1001, 1029.31 and 1037.89 MPa, respectively, and the maximum equivalent plastic strains are 0.205, 0.237 and 0.267, respectively. Compared with the C-shaped structure, the maximum stress of the U-shaped structure is reduced by 3.55%, and the maximum plastic strain is reduced by about 23%. The strain drop is significantly greater than the stress drop, indicating that the main advantage of the U-shaped design at the end-corner of the stent end is not to simply reduce the material bearing level, but to improve the deformation distribution in the bending area. The continuous arc makes the local rotation complete along a longer geometric path, which reduces the plastic strain concentration caused by the sharp geometric transition. Balloon-expandable stents need to undergo the whole process of ‘crimping-expansion-long-term cycle’. Suppression of strain concentration is critical, since cyclically loaded high-strain zones are more prone to fatigue-crack initiation than locations experiencing merely high peak stress. Therefore, the value of the U-shaped end structure is to consider both the expansion flexibility and the local fatigue safety reserve.

The results of different specifications of the stent further show that the mechanical response of the stent cannot be simply understood as ‘the larger the size, the greater the stress’. The four specifications are evenly pressed to 6.50 mm. As the initial diameter increases from 20 mm to 29 mm, the degree of crimping increases, and the maximum equivalent stress of crimping increases from 1030.5 MPa to 1076.1 MPa, indicating that a larger radial compression ratio will increase the structural bending and plastic deformation requirements. However, in the expansion stage, the maximum equivalent stress increased from 1417.3 MPa to 1492.5 MPa and then decreased to about 1455.4 MPa in the 29 mm specification. The maximum equivalent plastic strain also shows 0.447, 0.541, 0.544 and 0.510, rather than monotonically increasing with diameter. This result suggests that the local response of the stent is not only affected by the nominal diameter, but also depends on the mesh expansion angle, the degree of node rotation, and the plastic deformation history at different stages. Therefore, for a series of valve stents, it is not appropriate to obtain different specifications simply by geometric proportional amplification, but to evaluate the local mechanical state of each specification under the crimping and expansion paths, respectively. Mechanically, this decrease can be attributed to the path-dependent coupling between the common crimping step and the subsequent expansion step. Because all four stents were crimped to the same 6.50 mm diameter, the 29 mm stent underwent the largest radial compression ratio and entered the expansion step with a different residual plastic deformation, local curvature, and stress state. During expansion, diameter does not scale the local response linearly: struts rotate and straighten, axial foreshortening changes the load transfer path, and the critical corner may shift between neighboring nodes. The 26 mm specification appears to place the governing corner in a particularly unfavorable orientation, producing the peak local bending of 1492.5 MPa. In the 29 mm specification, the larger initial cell angle and greater pre-expansion rotation can redistribute deformation to adjacent struts and reduce the curvature and bending moment at the governing node. Part of the imposed expansion is therefore accommodated by plastic deformation and reorganization of the local load path rather than by a further increase in local von Mises stress. The reduction to 1455.4 MPa should thus be interpreted as a local redistribution effect associated with the deformation path, not as a reduction in the overall expansion demand or a monotonic size–strength relation. The corresponding decrease in maximum plastic strain from 0.544 to 0.510 supports this interpretation.

From the perspective of the deformation mechanism, the maximum equivalent plastic strain of the stent is 0.221~0.259 when the stent is crimped, and increases to 0.447~0.544 after expansion, and the high value continues to be concentrated in the end-corner area, indicating that the diameter of the balloon-expanded stent after maintenance release mainly depends on the irreversible plastic deformation in the connection area. In this study, there was no obvious structural damage to the solid stent after expansion, and the long-term fatigue test was completed subsequently, which provided support for the plastic deformation level at the engineering level.

Good correlation was observed between finite element predictions and bench-test measurements [26]. The overall contour and mesh deformation direction of the CDT07-20 and CDT07-26 solid stents after crimping and expanding are basically consistent with the simulation results [27]. In the recoil test of four specifications, the crimping recoil error is 2.56~5.88%, the expansion recoil error is 5.26~6.98%, and all absolute errors are no more than 0.06 mm. This shows that the model can not only reproduce the qualitative deformation mode of the stent but also predict the dimensional recovery behavior after unloading. As the specification increases, the amount of crimping and expansion recoil increases overall, reflecting that the large-size stent has a more obvious elastic recovery trend after experiencing greater radial deformation. Therefore, in the product design, not only should the final expansion diameter of the balloon be controlled, but also the recoil compensation after unloading should be considered in advance; otherwise, the actual stable diameter may be lower than the target release size.

Fatigue testing further addresses long-term in-service performance questions that static crimp-and-expand analyses cannot resolve. No obvious displacement or strut fracture was observed after 4 × 10^8^ cycles of fatigue testing in a PBS environment at (37 ± 2) °C, 20 Hz, and a pressure range of 80~160 mmHg—especially for the 29 mm specification, although it has the largest size and higher circumferential load level, indicating that the long-term reliability of the stent is not only determined by the overall size but is also closely related to the stress amplitude control at the joint and the load transfer mode of the composite mesh.

In summary, the structural design significance of this study is to form a design idea of ‘functional partition-local compliance-series size-whole process verification’: the composite mesh solves the functional requirements of different regions; the U-shaped end-corner improves local deformation transfer; 20~29 mm specifications cover different size requirements; and crimping, expansion, recoil, and fatigue evaluation together constitute a mechanical evidence chain from one-time large deformation to long-term cyclic service. At the same time, the existing results mainly verify the mechanical feasibility of the bare stent structure, which cannot be directly equivalent to the clinical suitability of the complete TAVI system [28]. There are also factors such as non-circular annulus [29], uneven calcified plaque [30], valve leaflet constraint, and coronary opening position differences after actual implantation. In the future, the contact pressure, anchoring stability, perivalvular leakage risk, and complete valve hemodynamic performance [31] should be further evaluated in the patient-specific aortic root model [32], to further promote the current structural mechanics optimization to the patient-specific instrument design and verification.

## 7. Conclusions

(1) The influence of stent structure and end-corner configuration on mechanical properties: In this study, the diamond–hexagonal composite mesh structure was selected from four preliminary schemes. Comparison of the three end-corner configurations shows that the maximum equivalent stress and the maximum equivalent plastic strain at the U-shaped end-corner are 1001 MPa and 0.205, respectively, both lower than those of the Y-shaped and C-shaped counterparts. This is attributed to the continuous arc transition to the U-shaped corner, which improves the load transfer path and reduces the local stress concentration caused by abrupt geometric changes. Therefore, the fillet and transition form at the end-corner are key structural parameters that influence the crimping and expansion performance as well as the durability of the stent.
(2) Crimping, expansion, and recoil behavior: Significant plastic deformation occurred after the four stent specifications were crimped to 6.50 mm, with the maximum equivalent plastic strain ranging from 0.221~0.259. Upon expanding to the target diameter, the maximum equivalent plastic strain increased to 0.447~0.544, and the high-strain regions were mainly concentrated at the end-corner of the stent. This indicates that the balloon-expandable stent primarily relies on the plastic deformation of the connection area to maintain its post-expansion shape; however, the elevated local plastic strain also suggests that these connections may become damage-sensitive zones. In the recoil analysis, the relative error between the simulation and the experiment was less than 7%, indicating that the model effectively predicts the overall radial recoil.
(3) Fatigue durability evaluation: After 4 × 10^8^ cycles of accelerated fatigue testing, no obvious strut fracture or surface damage occurred in the sample, indicating that the designed stent has good in vitro durability under the load and test conditions of this study.
(4) Clinical significance and research limitations: Small annular dimensions and the high prevalence of bicuspid aortic valves among Chinese patients were considered during size-series definition and mesh design, which provided a structural basis for the subsequent design of a domestic balloon-expandable valve stent. However, the current model has not yet included patient-specific aortic root, calcified plaque, valve leaflets, and coronary openings, and has not evaluated anchoring force, perivalvular leakage, and complete valve hemodynamic performance. Therefore, the current results mainly prove the mechanical feasibility and in vitro durability of the valve stent. Subsequent studies should combine patient-specific implant models, complete valve pulsation flow tests, the fatigue durability of the valve leaflets, and animal experiments to further verify safety and clinical suitability. Therefore, this study is limited to the mechanical feasibility and in vitro durability of the bare stent structure and should not be interpreted as validation of the complete TAVI system.

## Figures and Tables

**Figure 1:**
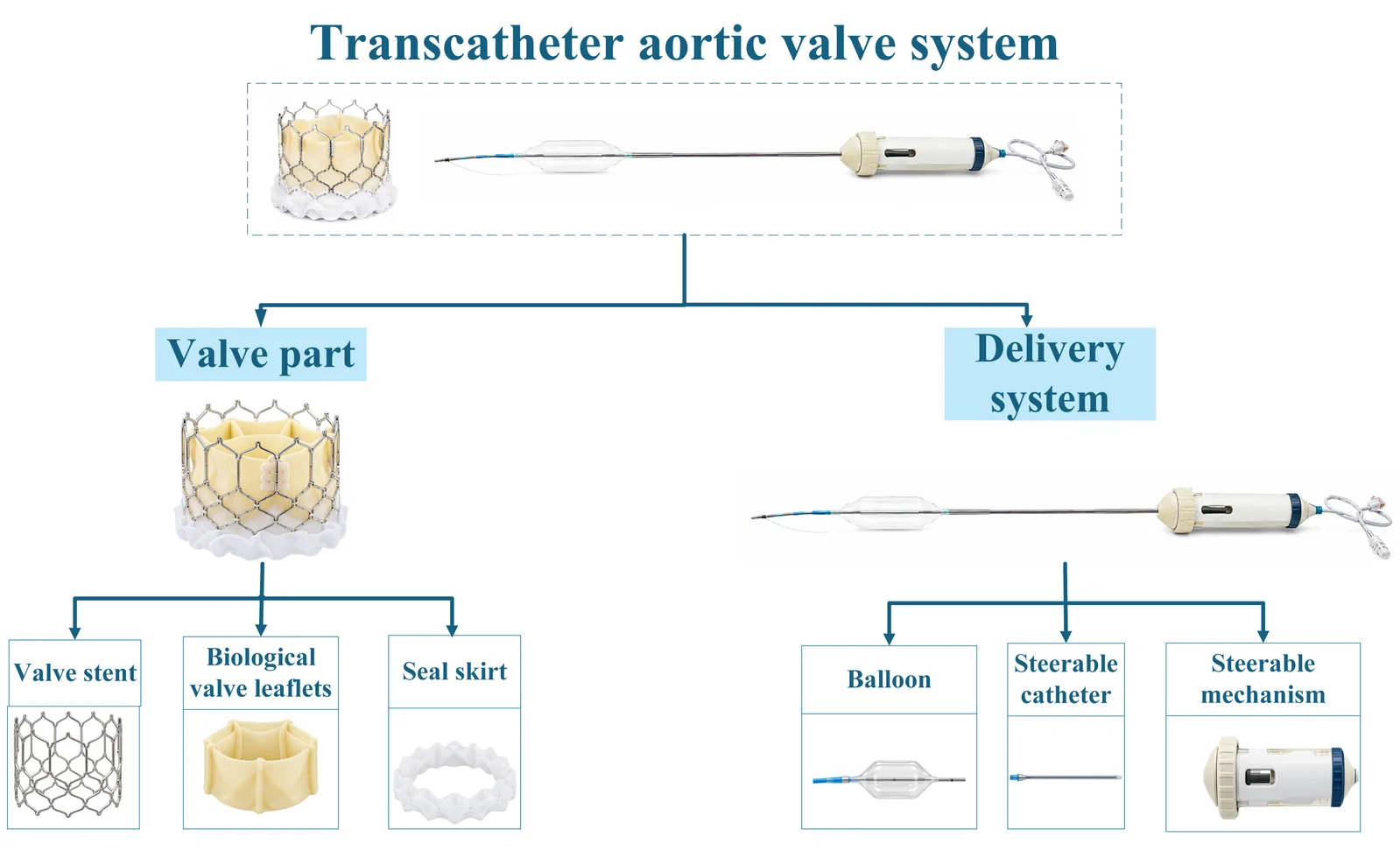
Transcatheter aortic valve system.

**Figure 2:**
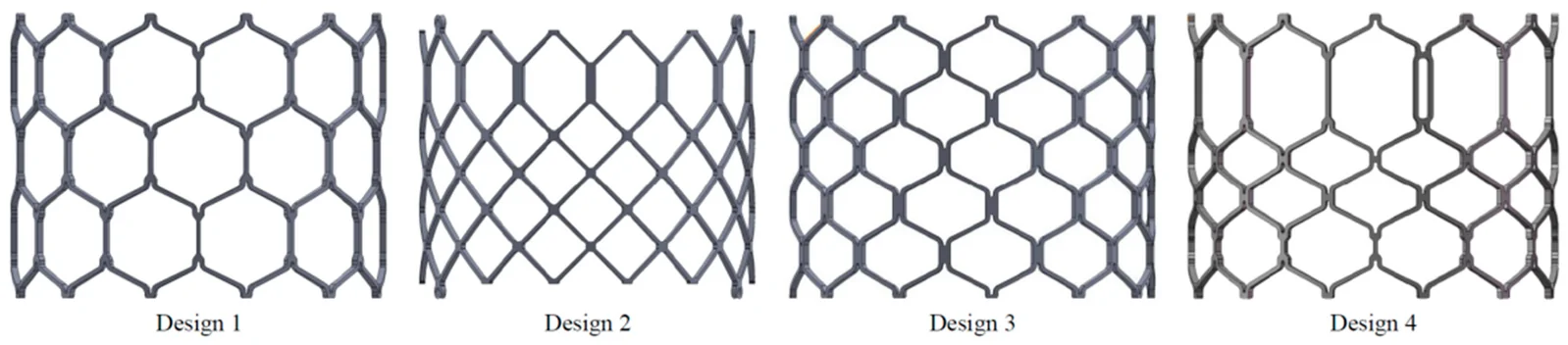
Four design schemes of valve stents.

**Figure 3:**
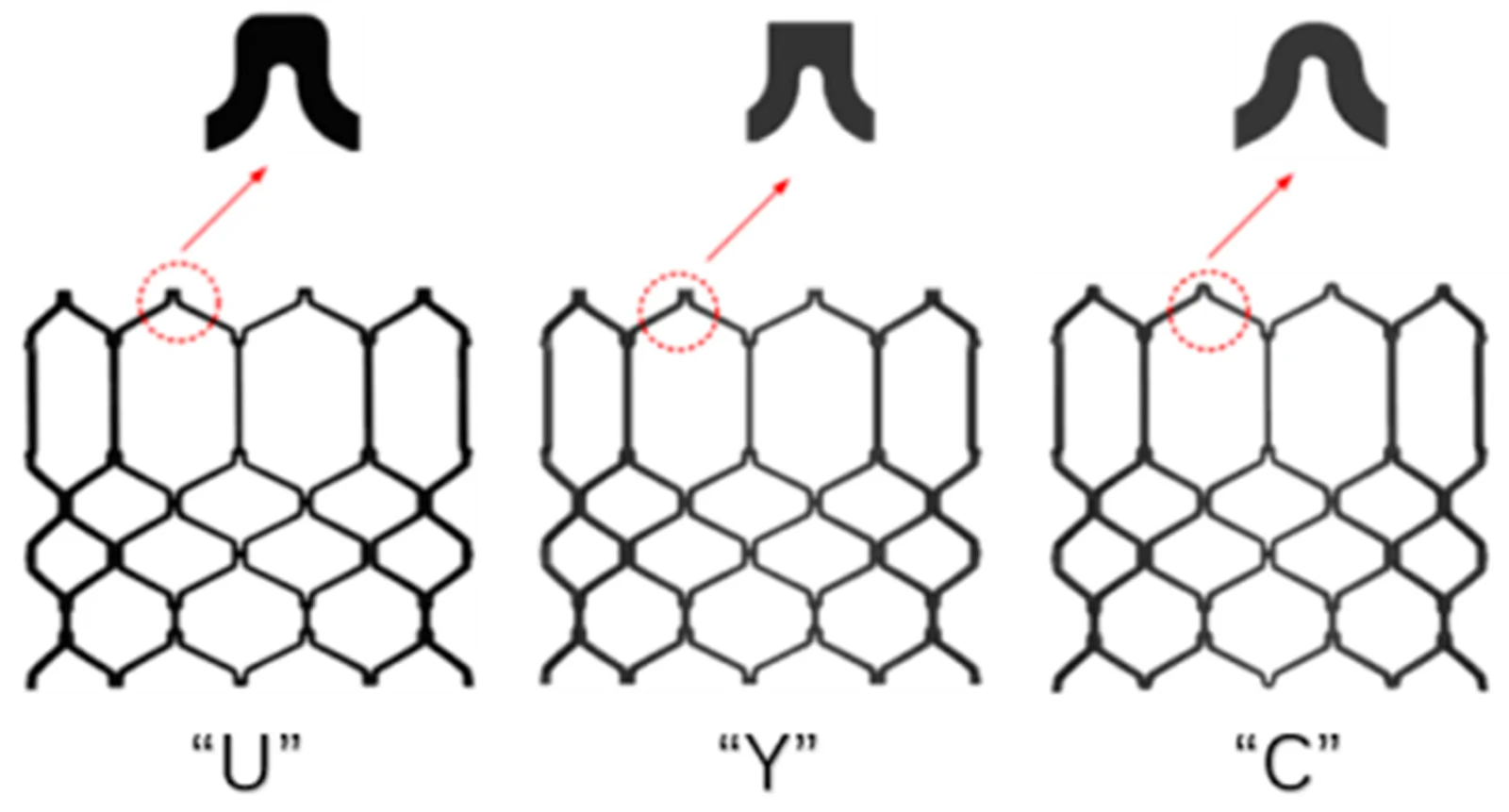
Three kinds of stent end-corner structures. (‘U’ shape, ‘Y’ shape, ‘C’ shape) schematic diagram.

**Figure 4:**
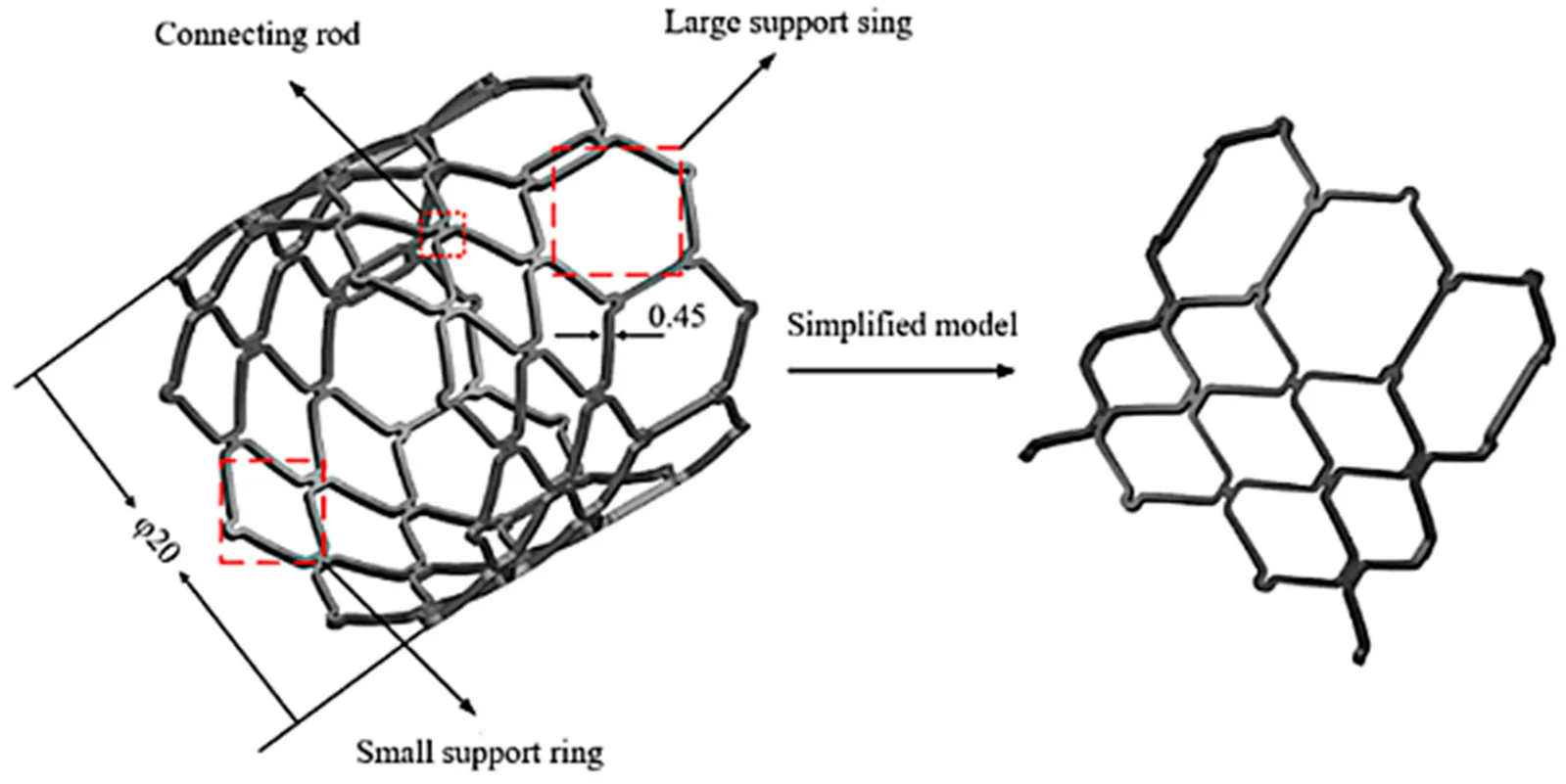
The overall model of the stent and the symmetric model for finite element analysis.

**Figure 5:**
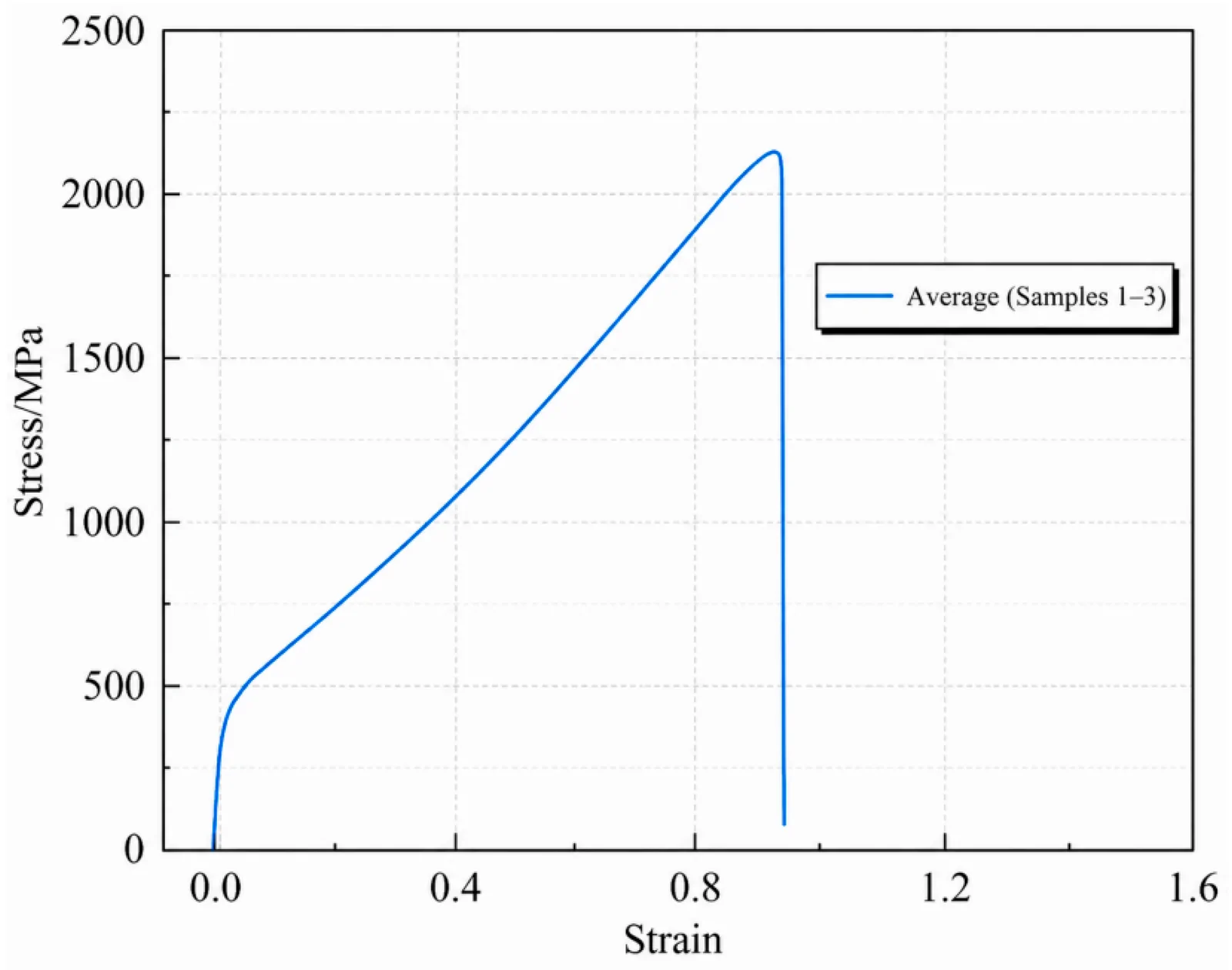
The true stress–strain curve of Co-Ni-Cr alloy MP35N.

**Figure 6:**
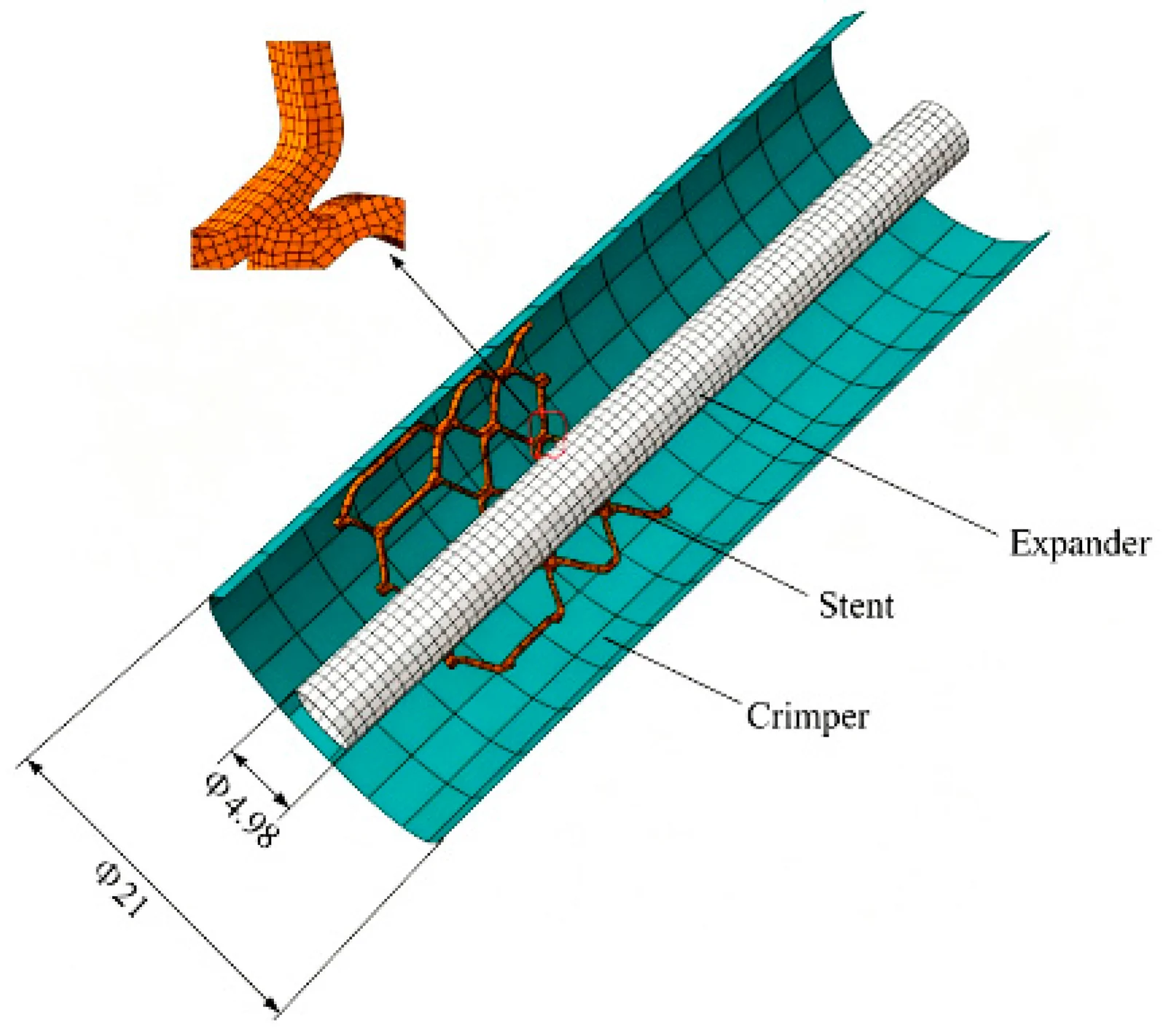
Finite element meshes of assembled stent and tools.

**Figure 7:**
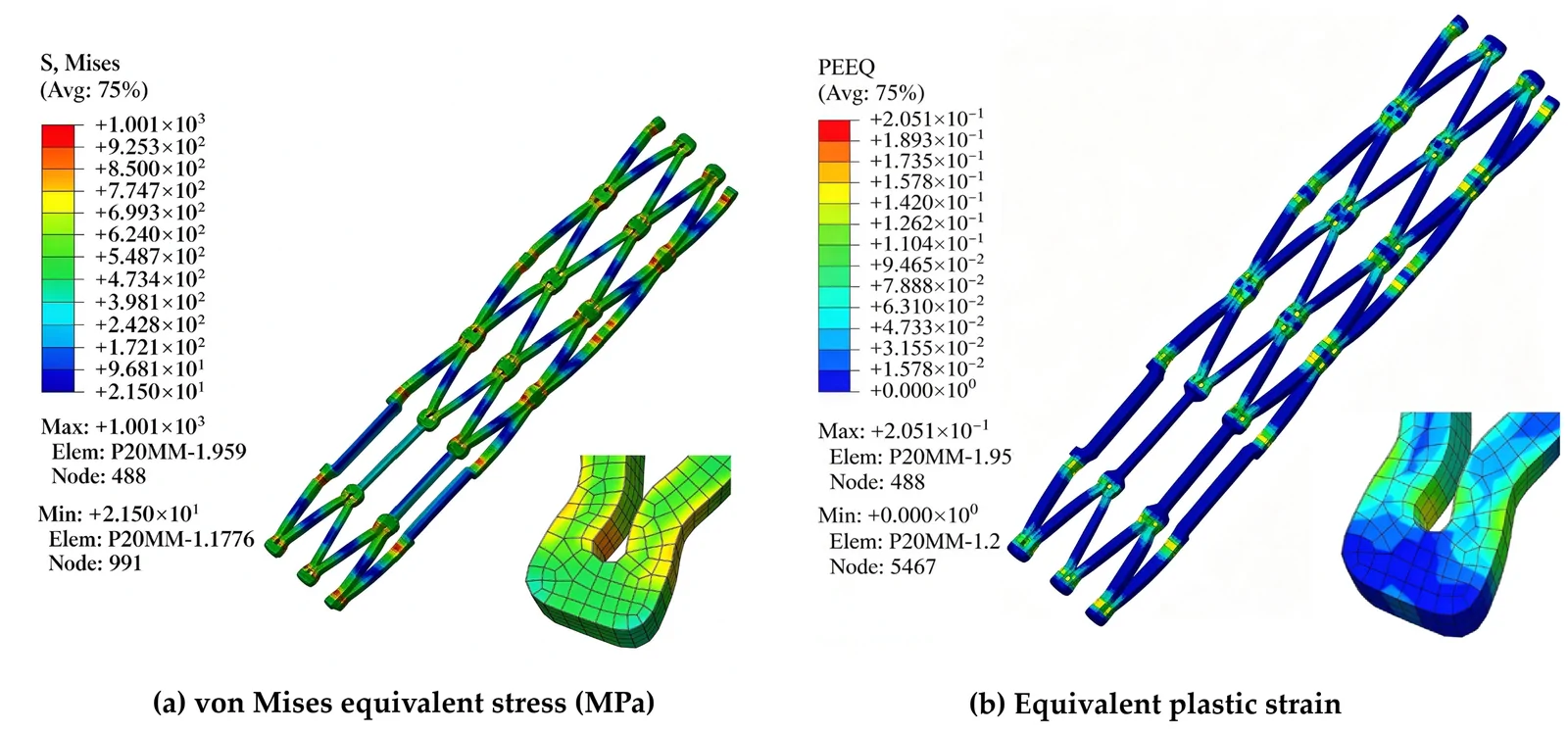
The crimp stress (**a**) and strain (**b**) of the CDT07-20 ‘U’-shaped stent.

**Figure 8:**
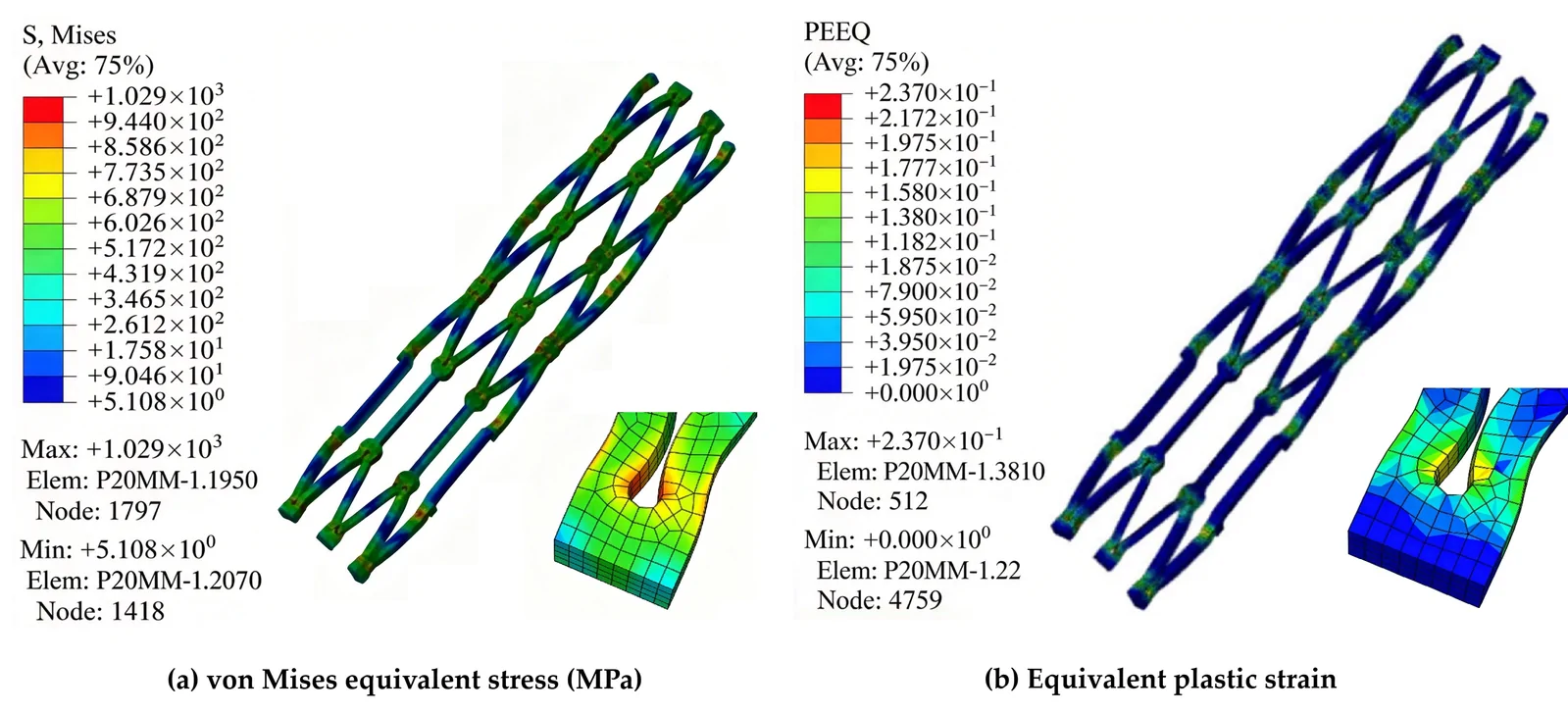
The crimp stress (**a**) and strain (**b**) of the CDT07-20 ‘Y’-shaped stent.

**Figure 9:**
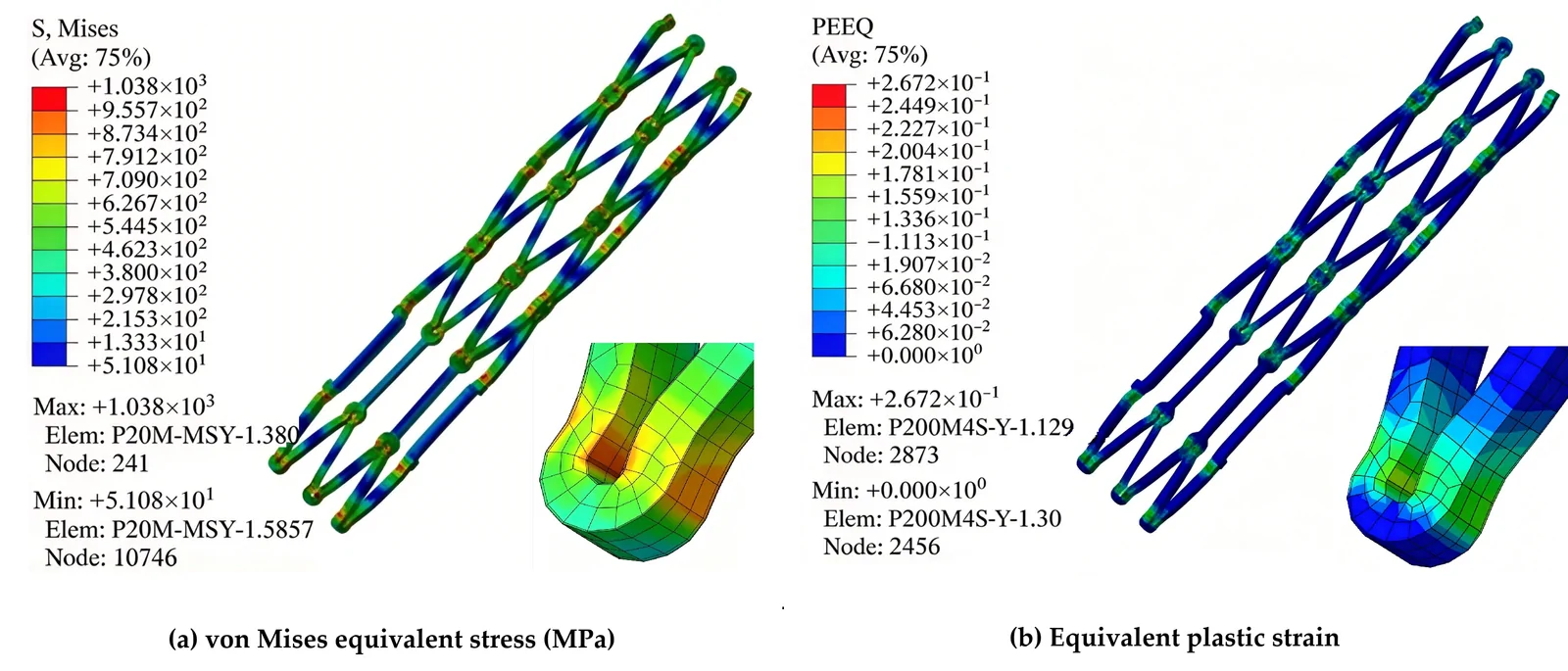
The crimp stress (**a**) and strain (**b**) of the CDT07-20 ‘C’-shaped stent.

**Figure 10:**
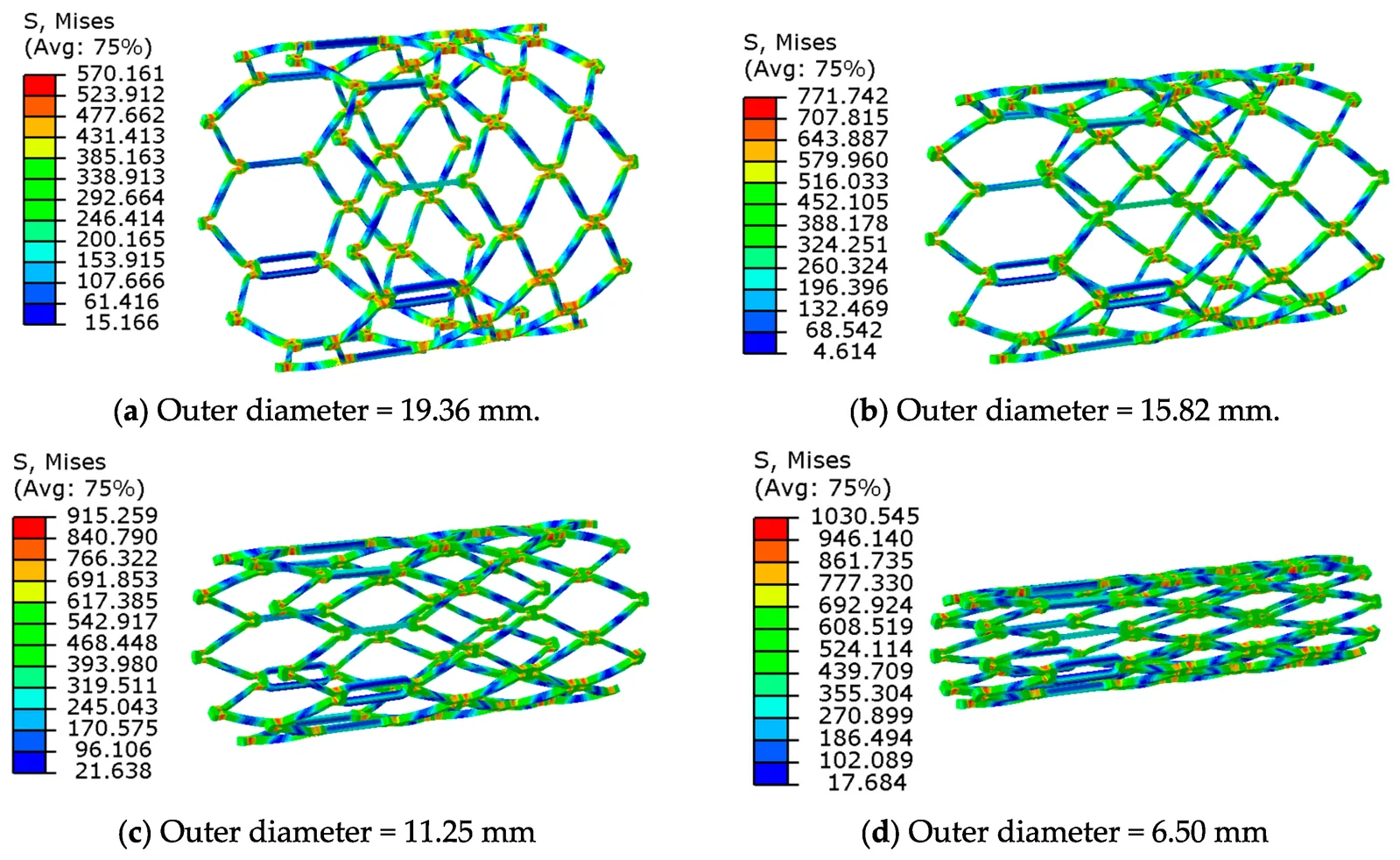
Equivalent stress distribution (MPa) of valve stent subjected to radial compression.

**Figure 11:**
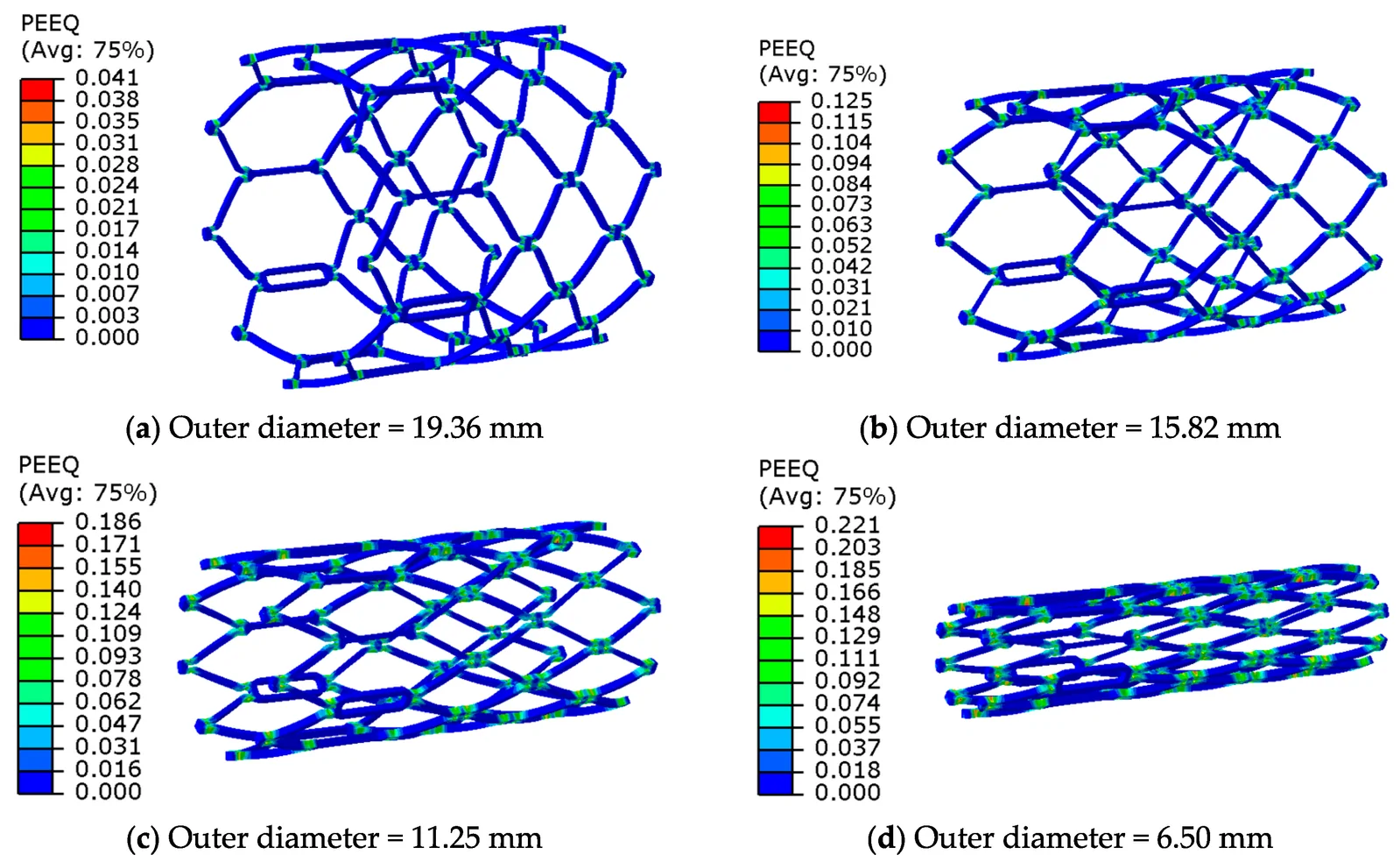
Equivalent plastic strain distributions of valve stent subjected to radial compression.

**Figure 14:**
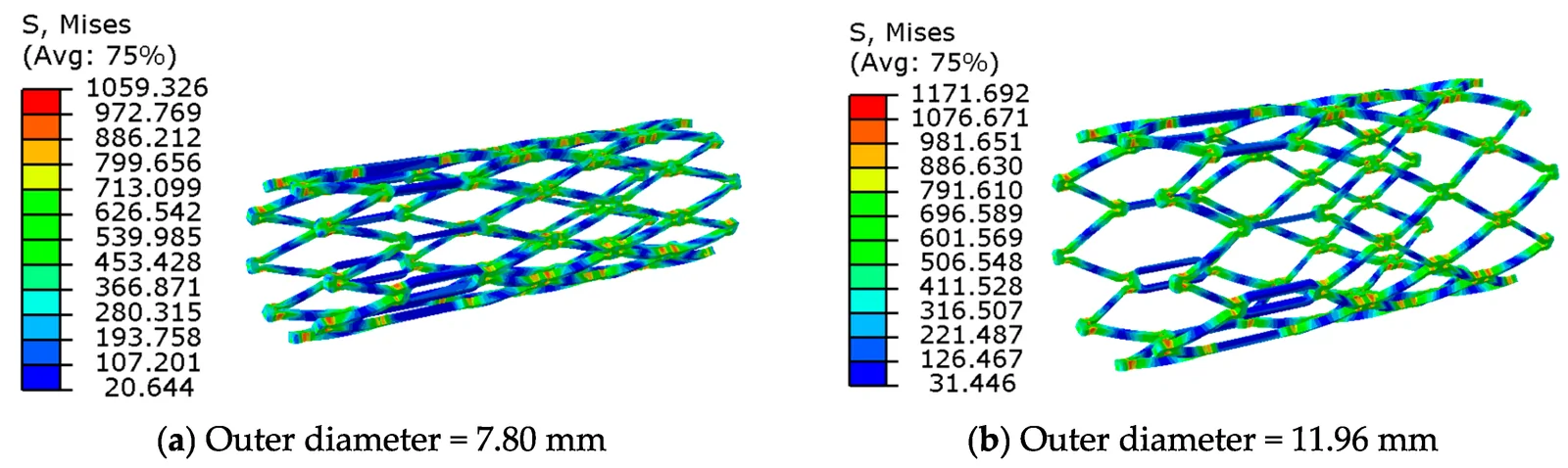

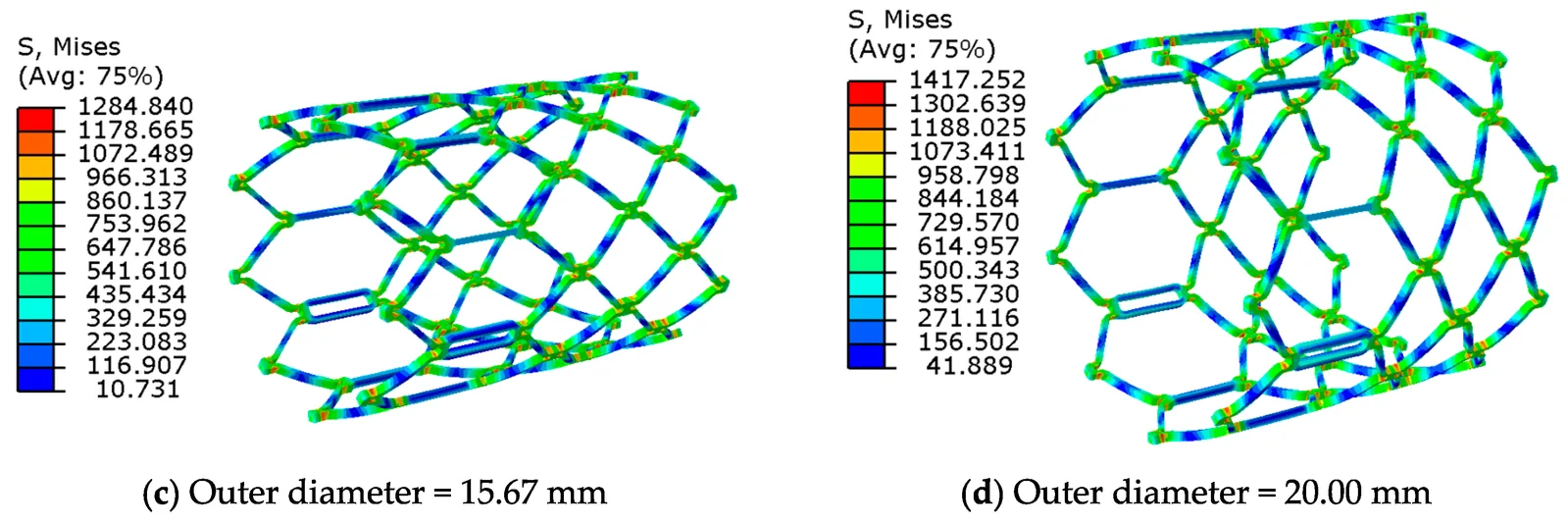
The equivalent stress distribution (MPa) of the valve stent subjected to radial expansion.

**Figure 15:**
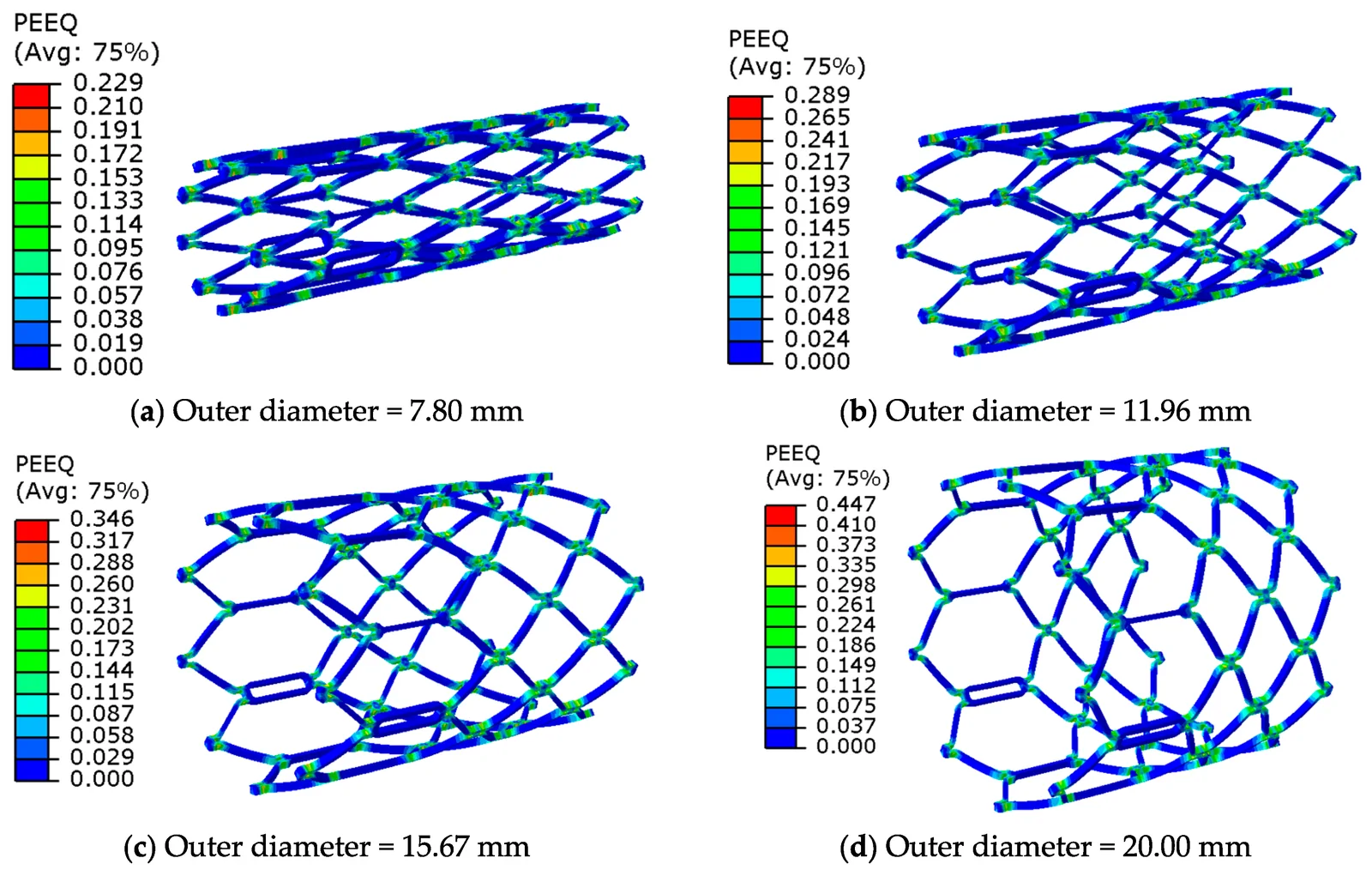
Equivalent plastic strain distribution of valve stent with radial expansion.

**Figure 16:**
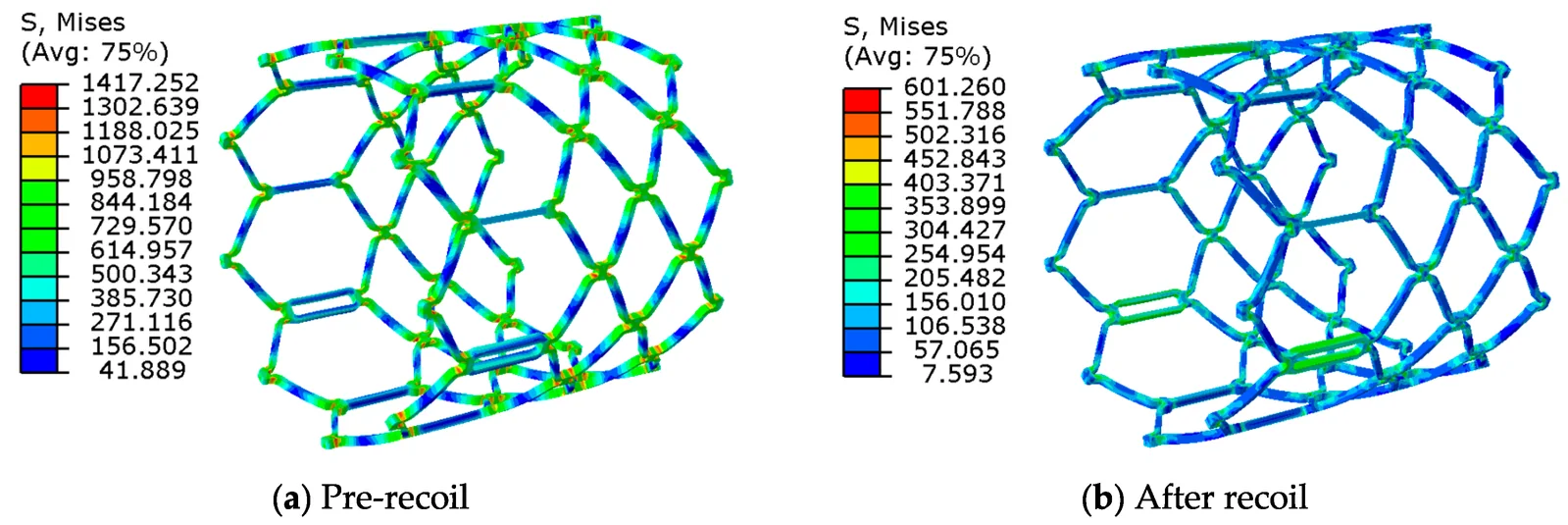
The residual stress distribution (MPa) before and after expansion and recoil of the valve stent.

**Figure 17:**
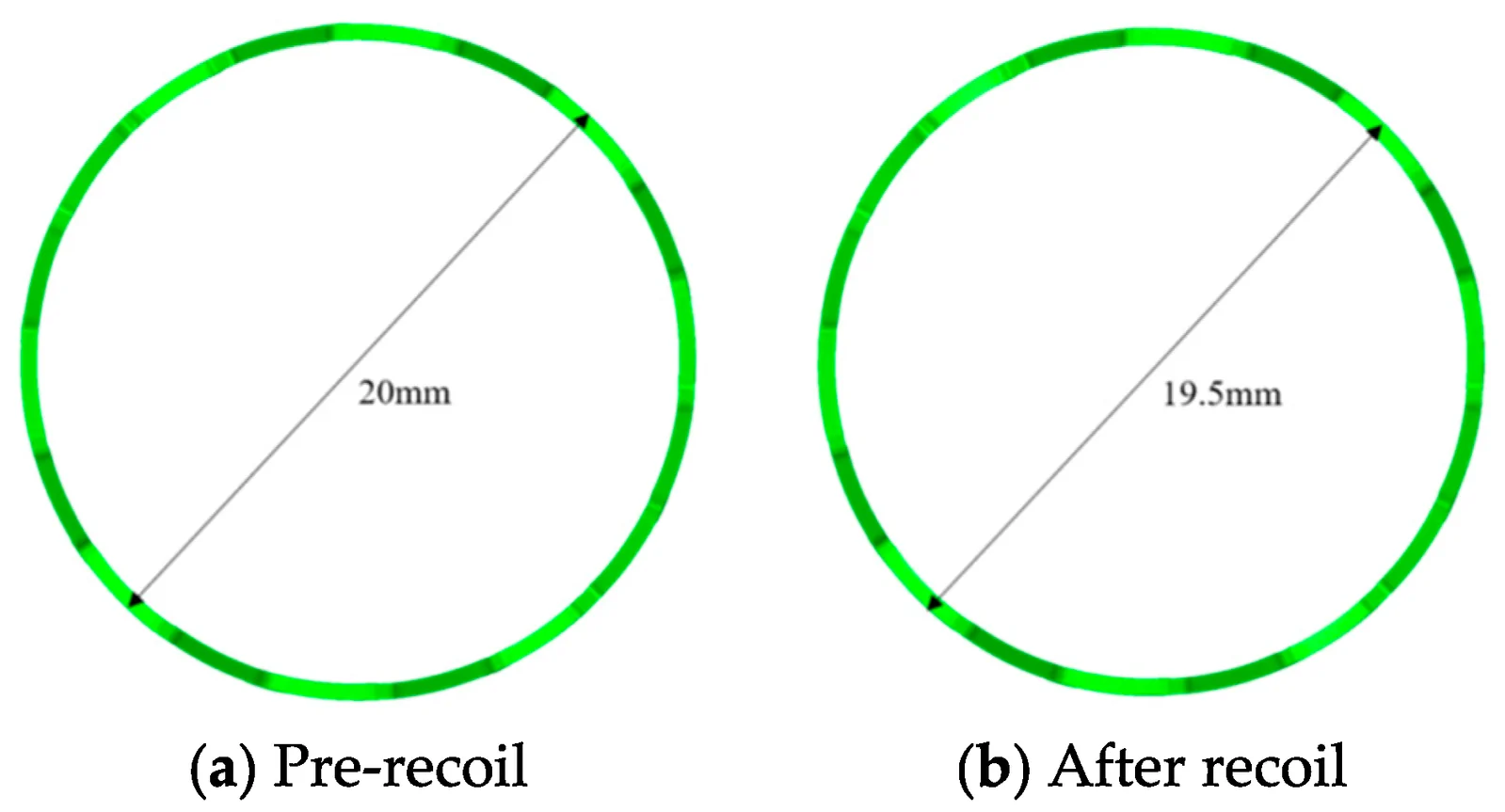
The external diameter of the valve stent before and after expansion recoil.

**Figure 18:**
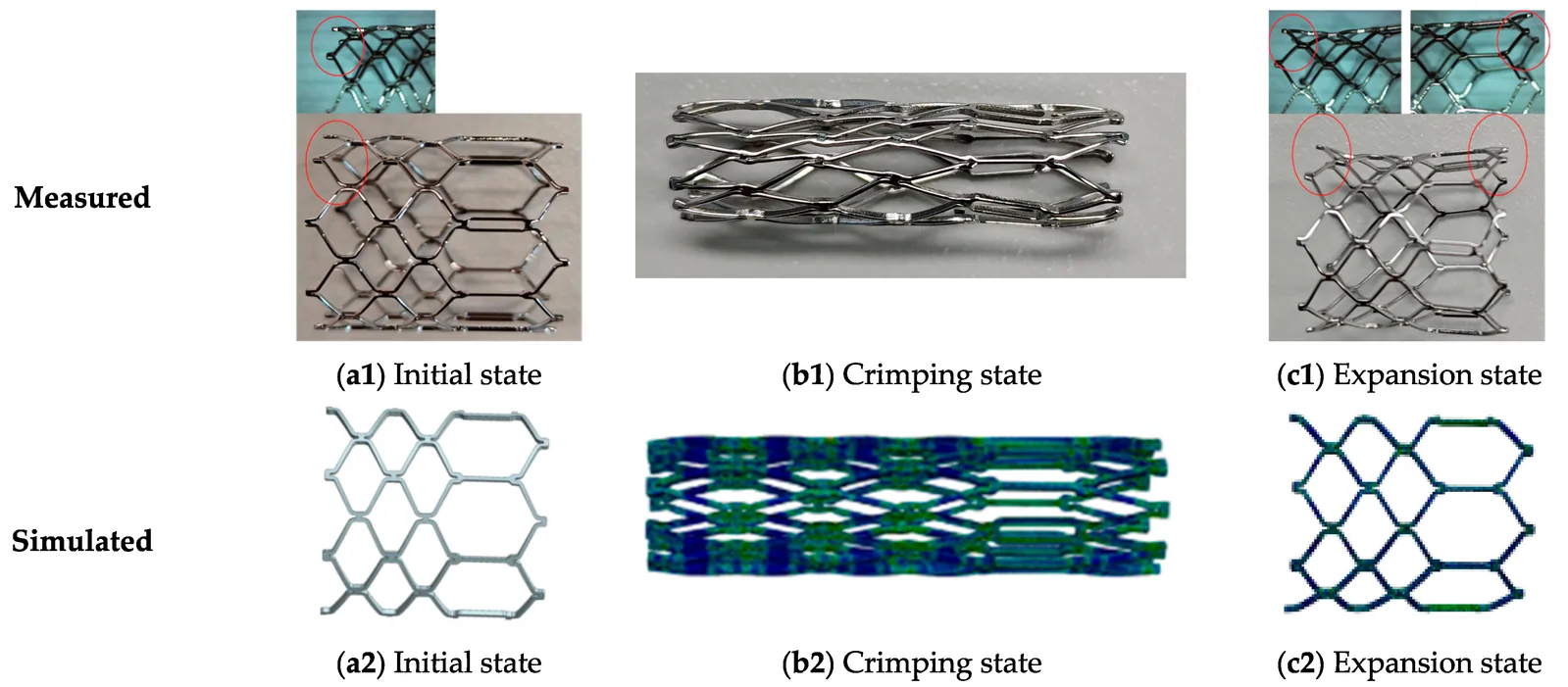
Measurement and simulation of three states of the CDT07-20 specification stent model.

**Figure 19:**
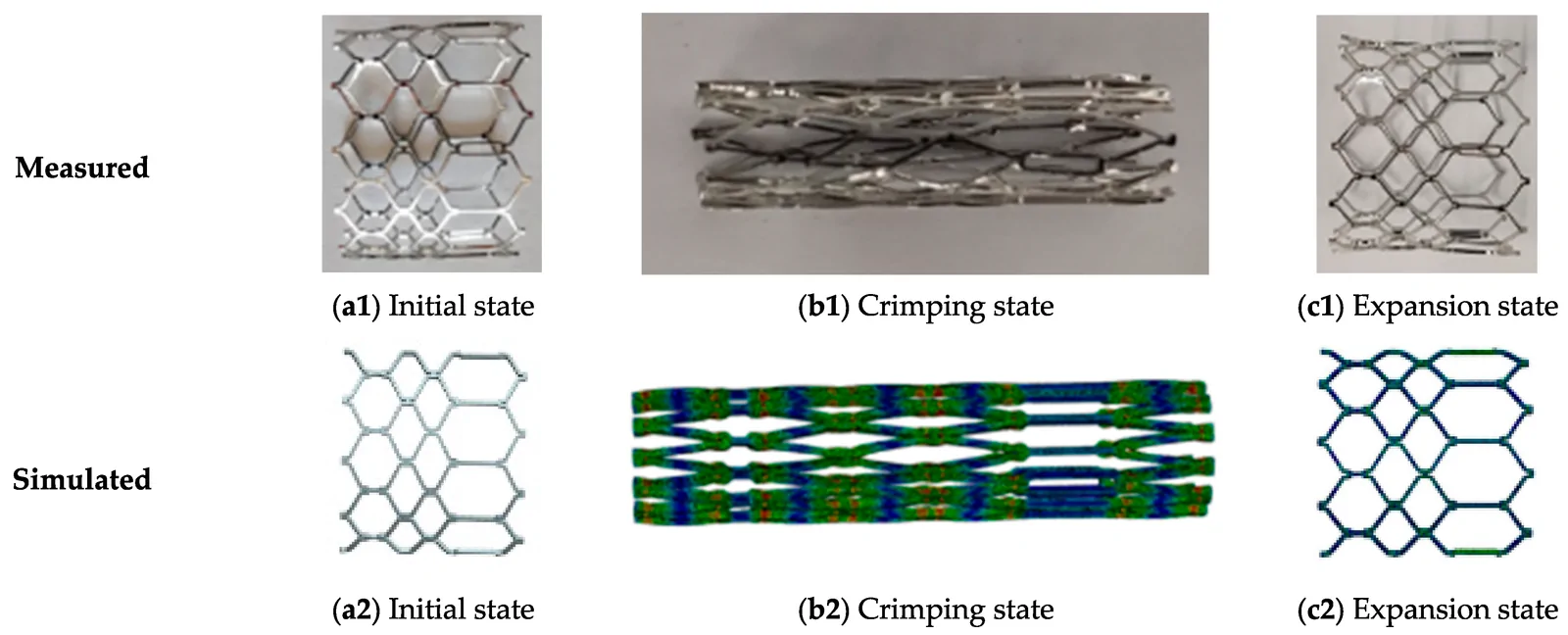
Measurement and simulation of three states of CDT07-26 specification stent model.

**Figure 20:**
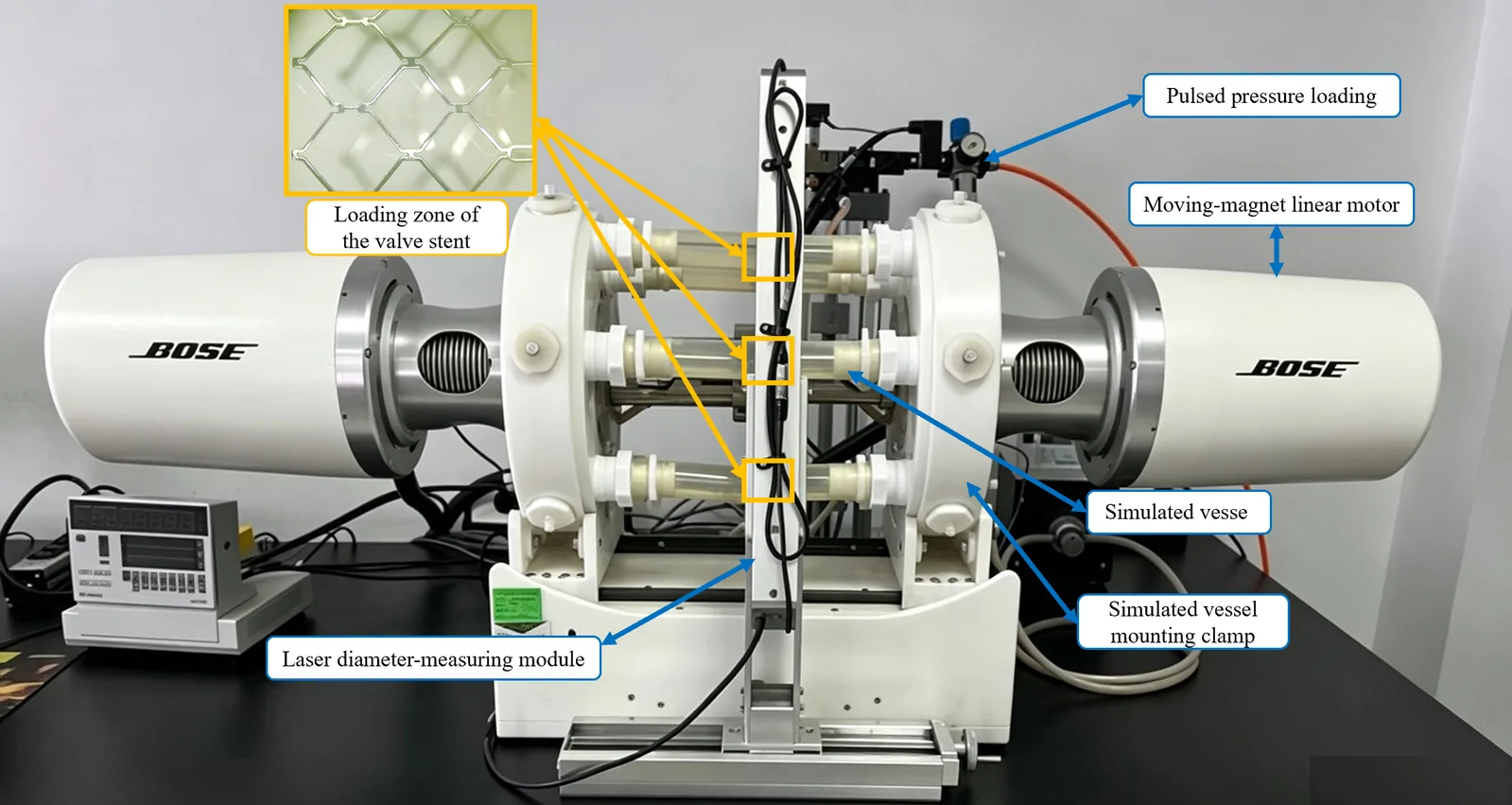
Fatigue test of stent.

**Figure 21:**
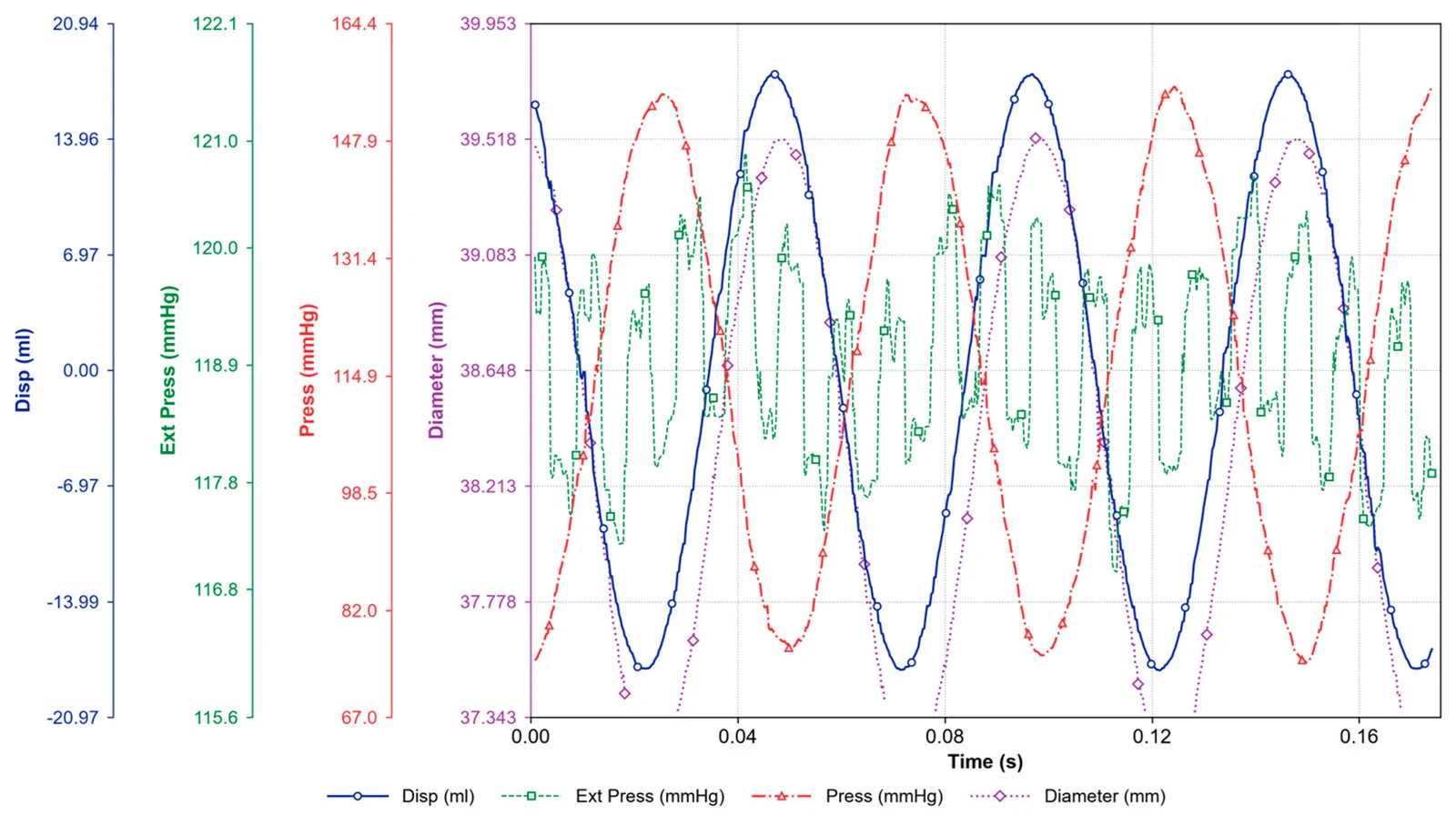
A waveform diagram of the stent fatigue durability test (local).

**Figure 22:**
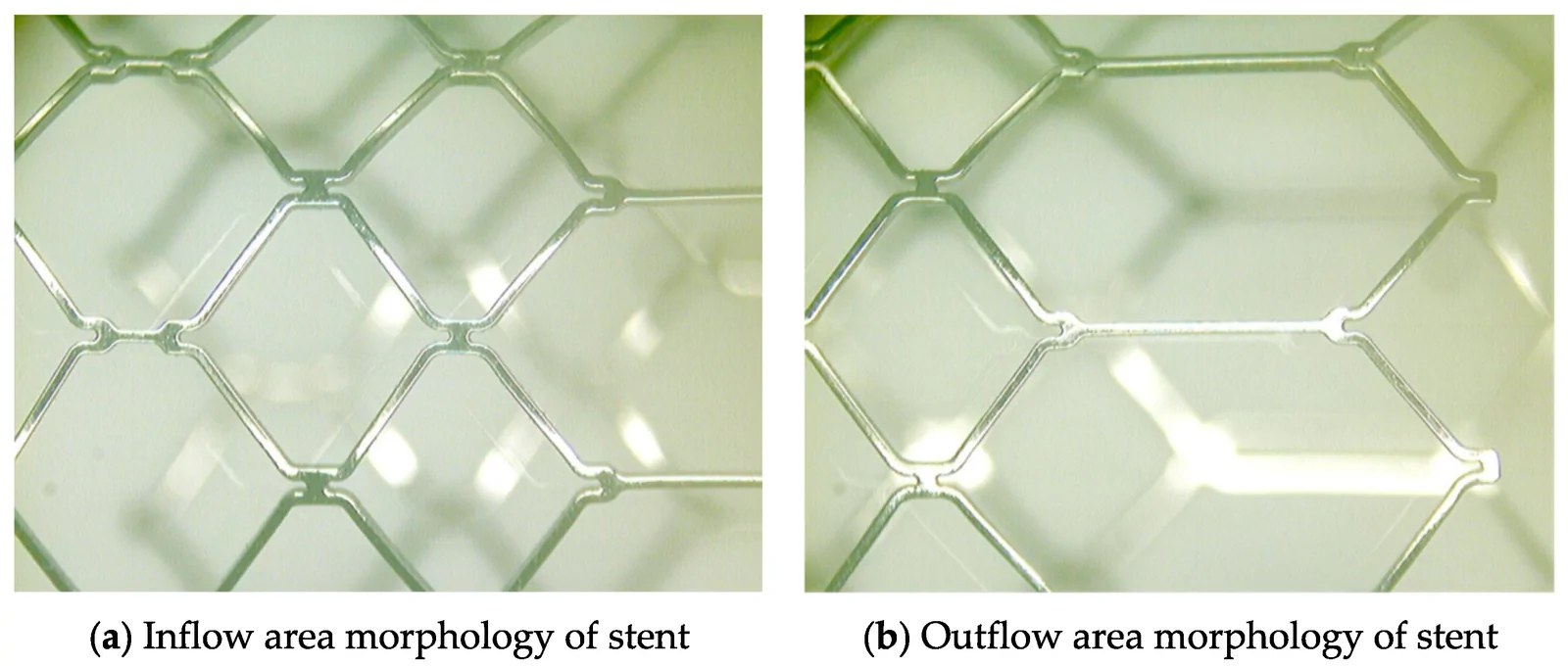
The durability test morphology of the valve stent.

**Table 1:** The simulated maximum equivalent stress, the maximum equivalent plastic strain, and the outer diameter after recoil of the valve stent at each stage.

Specifications	Maximum Equivalent Stress (MPa)				Maximum Equivalent Plastic Strain		Recoil After Deformation (mm)	
	Crimping	Recoil After Crimping	Expansion	Recoil After Expansion	Crimping	Expansion	Crimping	Expansion
CDT07-20	1030.5	479.5	1417.2	601.2	0.221	0.447	7.3	19.5
CDT07-23	1032.1	520.5	1483.9	664.9	0.242	0.541	7.4	22.4
CDT07-26	1045.9	643.9	1492.5	591.9	0.259	0.544	7.6	25.3
CDT07-29	1076.1	635. 4	1455.4	580.5	0.248	0.510	7.8	28.2

**Table 2:** Comparison of recoil ratio of stents with various specifications: test vs. simulation.

Specification	The Amount of Crimping Recoil/mm		Crimping Recoil Error	The Amount of Expansion Recoil/mm		Expansion Recoil Error
	Simulated	Measured		Simulated	Measured	
CDT07-20	0.80	0.78	2.56%	0.50	0.47	6.38%
CDT07-23	0.90	0.85	5.88%	0.60	0.57	5.26%
CDT07-26	1.10	1.04	5.77%	0.70	0.74	5.41%
CDT07-29	1.30	1.23	5.69%	0.80	0.86	6.98%

## Data Availability

The original contributions presented in this study are included in the article. Further inquiries can be directed to the corresponding author.
